# Cyanobacterial photosynthesis under sulfidic conditions: insights from the isolate *Leptolyngbya* sp. strain hensonii

**DOI:** 10.1038/ismej.2017.193

**Published:** 2018-01-12

**Authors:** Trinity L Hamilton, Judith M Klatt, Dirk de Beer, Jennifer L Macalady

**Affiliations:** 1 Department of Biological Sciences, University of Cincinnati, Cincinnati, OH 45221, USA; 2 Max-Planck Institute for Marine Microbiology, Bremen, Germany; 3 Geomicrobiology Lab, Department of Earth and Environmental Sciences, University of Michigan, Ann Arbor, MI, USA; 4 Department of Geosciences and the Penn State Astrobiology Research Center (PSARC), Pennsylvania State University, University Park, PA 16802, USA

## Abstract

We report the isolation of a pinnacle-forming cyanobacterium isolated from a microbial mat covering the sediment surface at Little Salt Spring—a flooded sinkhole in Florida with a perennially microoxic and sulfidic water column. The draft genome of the isolate encodes all of the enzymatic machinery necessary for both oxygenic and anoxygenic photosynthesis, as well as genes for methylating hopanoids at the C-2 position. The physiological response of the isolate to H_2_S is complex: (i) no induction time is necessary for anoxygenic photosynthesis; (ii) rates of anoxygenic photosynthesis are regulated by both H_2_S and irradiance; (iii) O_2_ production is inhibited by H_2_S concentrations as low as 1 μM and the recovery rate of oxygenic photosynthesis is dependent on irradiance; (iv) under the optimal light conditions for oxygenic photosynthesis, rates of anoxygenic photosynthesis are nearly double those of oxygenic photosynthesis. We hypothesize that the specific adaptation mechanisms of the isolate to H_2_S emerged from a close spatial interaction with sulfate-reducing bacteria. The new isolate, *Leptolyngbya* sp. strain hensonii, is not closely related to other well-characterized Cyanobacteria that can perform anoxygenic photosynthesis, which further highlights the need to characterize the diversity and biogeography of metabolically versatile Cyanobacteria. The isolate will be an ideal model organism for exploring the adaptation of Cyanobacteria to sulfidic conditions.

## Introduction

Cyanobacteria are the only chlorophototrophs that carry out oxygenic photosynthesis and presumably provided the first significant source of O_2_ on early Earth. The evolution of oxygenic photosynthesis in ancient Cyanobacteria transformed Earth, ultimately providing conditions that ushered in complex multicellular life forms. However, even after the first global rise of atmospheric O_2_ during the Great Oxidation Event, concentrations in ocean surface waters remained low and sulfidic conditions were common throughout much of the Proterozoic (Canfield, 1998; Meyer and Kump, 2008), particularly in restricted basins and along productive continental margins (Scott *et al.*, 2008; Lyons *et al.*, 2009; Poulton *et al.*, 2010; Poulton and Canfield, 2011). Multiple phylogenetic analyses suggest that the less complex, anoxygenic modes of photosynthesis evolved before oxygenic photosynthesis (Blankenship, 2001; Xiong and Bauer, 2002; Sadekar *et al.*, 2006; Bryant and Liu, 2013). Anoxygenic phototrophs use one reaction center, which may be either type 1 or type 2, and do not evolve oxygen. Anoxygenic photosynthesis relies on a supply of reducing equivalents from reduced sulfur compounds, organic acids, hydrogen, nitrite, arsenite or Fe(II) to drive CO_2_ reduction. Despite the ubiquity of H_2_O as an electron donor for oxygenic photosynthesis, observations of extant Cyanobacteria capable of performing anoxygenic photosynthesis have been documented in environments where sulfide is present in the photic zone and in a handful of pure cultures (Cohen *et al.*, 1975a, 1975b; Padan, 1979; de Wit and van Gemerden, 1987; Garcia-Pichel and Castenholz, 1990; Klatt *et al.*, 2015a).

The capability to perform anoxygenic photosynthesis has been considered a relic of cyanobacterial ancestors living before the evolution of oxygenic photosynthesis (Oren *et al.*, 1977; Padan, 1979). An alternative hypothesis is that photosynthetic versatility in Cyanobacteria represents an intermediate state during the evolution and fine-tuning of oxygenic photosynthesis, in which Cyanobacteria used either H_2_O or H_2_S in sulfidic photic zones such as those present at continental margins throughout most of Earth’s history (Hamilton *et al.*, 2016). In extant Cyanobacteria, the ability to perform anoxygenic photosynthesis, while not common, is widespread among phylogenetically diverse Cyanobacteria (Miller and Bebout, 2004). To date, all characterized Cyanobacteria that perform anoxygenic photosynthesis encode a sulfide quinone oxidoreductase (SQR), which oxidizes sulfide to sulfur and transfers electrons to photosystem I (PSI) (Arieli *et al.*, 1994; Theissen *et al.*, 2003). SQRs can also have other functions including sulfide detoxification. There is evidence that SQRs have been transferred horizontally (Theissen *et al.*, 2003), and genes encoding multiple types of SQRs are often found within the same genome. For SQR sequences in general, there is only rough correlation between the topology of phylogenetic trees of 16S rRNA and SQR sequences from the same organism (Pham *et al.*, 2008). The widespread taxonomic distribution of Cyanobacteria capable of performing anoxygenic photosynthesis in the absence of detectable heritability of this trait supports the emergence of this physiology multiple times through horizontal gene transfer. Still, these observations do not discern if SQR, or more specifically, anoxygenic photosynthesis, is an ancestral trait in Cyanobacteria.

Regardless, little of the physiology and ecology of ancient Cyanobacteria can be gleaned from the fossil record. Today Cyanobacteria are key primary producers in laminated mats of varying morphologies. Sulfur cycling is crucially important in these mats, which also host sulfate-reducing organisms and other anoxygenic phototrophs. Similar communities dominated by Cyanobacteria are thought to have been present in ancient stromatolites (Walter, 1976). Other fossil information including lipid, chlorophyll, and carotenoid biomarkers provide clues about ancient microbial community structure and physiology, but their interpretation is complicated by the diverse organisms that produce them. For instance, hopanoids methylated at the C-2 position were originally thought to be synthesized exclusively by Cyanobacteria (Summons *et al.*, 1999). More recent studies indicate that other organisms also produce these lipids, including anoxygenic phototrophs in proteobacterial clades (Rashby *et al.*, 2007; Welander *et al.*, 2010). The study of extant Cyanobacteria that make stromatolitic structures and lipid biomarkers under microoxic, sulfidic conditions may yield the insights necessary to interpret biosignatures in the rock record as well as to understand the physiology and ecology of ancient Cyanobacteria.

Cyanobacterial isolates of known purity capable of anoxygenic photosynthesis are rare (Cohen *et al.*, 1975a,1975b; de Wit and van Gemerden, 1987; Klatt *et al.*, 2015a) and the regulation of photosynthetic modes appears to vary among them (Cohen *et al.*, 1975a,1975b; Klatt *et al.*, 2015a). Biochemical characterization so far has only identified a single enzyme needed for H_2_S-driven anoxygenic photosynthesis: a SQR, providing electrons to PSI via the plastoquinone pool (PQ). Recent observations suggest that the light-independent enzyme kinetics of SQR control the rates of anoxygenic photosynthesis in Cyanobacteria when the sulfide concentration is low, whereas at higher levels of sulfide, light intensity dictates the upper limit of anoxygenic photosynthesis rates (Klatt *et al.*, 2015a, 2016a). These observations are, however, complicated by the variability of specific adaptations to fluctuating sulfide concentrations and irradiance in the environment, particularly in microbial mats, and by our lack of understanding of the mechanism of sulfide inhibition of PSII (Garcia-Pichel and Castenholz, 1990; Klatt *et al.*, 2015b). The affinity of SQR to both H_2_S and PQ, for instance, varies substantially among the few studied Cyanobacteria (de Wit and van Gemerden, 1987; Castenholz *et al.*, 1991; Griesbeck *et al.*, 2000; Klatt *et al.*, 2015a, 2016a). Overall, there are many gaps in our understanding of how environmental factors interact to determine the balance of oxygenic and anoxygenic photosynthesis in metabolically versatile Cyanobacteria.

Little Salt Spring (LSS), a flooded sinkhole in Florida, hosts a seasonal bloom of red pinnacle mats dominated by Cyanobacteria and green sulfur bacteria (GSB) (Hamilton *et al.*, 2017). The water column is sulfidic and despite the abundance of Cyanobacteria in the mat, only a small increase in oxygen (0.2 μM) has been observed in the water column during midday (de Beer *et al.*, 2017). The mat contains abundant hopanoids, including a significant fraction methylated at the C-2 position (Hamilton *et al.*, 2017). Here, we report the isolation and draft genome of a metabolically versatile, pinnacle-forming cyanobacterium from LSS. We examined whether the isolate is closely related to other Cyanobacteria capable of both types of photosynthesis and if the isolate could be the source of 2-methyl hopanoids in the pinnacle mats. We used microsensors to determine if the isolate can perform both types of photosynthesis, and developed a model of photosynthetic electron transport to explore potential regulatory mechanisms.

## Materials and methods

### Sample collection

Red pinnacle mat samples were collected by divers in June of 2012 from LSS, a 78-m diameter sinkhole lake located in Sarasota County, FL (lat. 27°04′30″N, long. 82°14′00″W). The geology and hydrology of the sinkhole have been described previously (Zarikian *et al.*, 2005) as well as the spring geochemistry and microbiology of the red pinnacle mat (Hamilton *et al.*, 2017). Diver-collected pinnacle mats from the water-sediment interface at ~14-m were placed into sterile 50-ml conical tubes, overlaid with spring water collected from the same location as the mat sample, and stored in the dark at 4 °C. Spring water was also collected at the water-sediment interface with syringes and immediately analyzed for sulfide concentration as they were brought to the surface. Dissolved sulfide concentration was measured with a portable spectrophotometer (Hach Co., Loveland, CO, USA), using methylene blue for total sulfide (Hach method 690, detection limit ~0.2 μM). Sulfide analyses were conducted in duplicate and were within 5% of each other. The total dissolved sulfide concentration was 21.6 μM at the mat–sediment interface at the time of sample collection. Photosynthetically active radiation (PAR) was measured at the surface of the red pinnacle mat at the time of collection using a scalar quantum PAR sensor (LiCor LI-193S) attached to a LI-COR LI-1400 data logger (LI-COR Biosciences, Lincoln, NE, USA). PAR at the surface of the red pinnacle mat was 65 μmol photons m^−2^ s^−1^ at the time of sample collection. Dissolved oxygen was measured *in situ* using a mulitparameter YSI 6600 sonde (YSI Inc., Yellow Springs, OH, USA) with a luminescent-based dissolved oxygen sensor (ROX). The sonde was calibrated daily according to the manufacturer’s protocol and a detection limit of 0.3 μM was determined for the ROX sensor. In addition, *in situ* oxygen microsensor measurements were recorded in November of 2014 (de Beer *et al.*, 2017).

### Enrichment, isolation and microscopy

Samples of red pinnacle mat were homogenized and a small aliquot (~500 μl) was added to BG11 media (Rippka *et al.*, 1979) supplemented with 25 mM HEPES (B-HEPES) and adjusted to pH 7.2. Enrichment cultures were maintained in 60 ml of liquid media in 125-ml conical flasks at 100 r.p.m. at 28 °C under either a day–night cycle or continuously illuminated with 100 μmol photons m^−2^ s^−1^ under cool white fluorescent lamps. An axenic culture was achieved using a dilution series in liquid media where the highest dilution that showed growth was taken as the inoculum for the next dilution. Light microscopy was performed periodically to visually examine enrichments for purity. The dilution to extinction strategy was continued until light microscopy indicated the presence of a single morphotype and sequencing of the 16S rRNA gene returned a single phylotype. In addition, to test for heterotrophic contaminants, samples of the axenic culture were plated on LB agar medium supplemented with 1% tryptone and 0.5% yeast extract. No colonies were observed on the plates after incubation in the dark for 5 days. Growth of the isolate was monitored with chlorophyll *a* concentration determined spectrophotometrically using the absorption at 665 nm of a methanol extract and an extinction coefficient of 0.075 ml μg^−1^ (made from a filtered 2-ml culture subsample) (De Marsac and Houmard, 1988) or protein concentration using the Bradford assay (Bradford, 1976) with bovine serum albumin (Sigma-Aldrich, St Louis, MO, USA) as the standard. The isolate is filamentous and forms biofilms and pinnacles.

The isolate was imaged using using an Olympus BX53 digital microscope (Olympus, Tokyo, Japan) and an Olympus DP73 digital camera with Cellsens digital image software (Olympus American Inc., Center Valley, PA, USA).

### Nucleic acid analyses

Samples of biofilm (~1.5 ml) were harvested by centrifugation, the excess media removed by decanting and cell pellets frozen immediately (−20 °C) or subjected to nucleic acid extraction. Genomic DNA was extracted as described previously (Boyd *et al.*, 2007). Quality of extracted DNA was assessed on an agarose gel (1%) using the HiLo DNA Marker (Bionexus, Oakland, CA, USA) and visualized by ethidium bromide staining and using a NanoDrop ND-1000 spectrophotometer (NanoDrop Technologies, Wilmington, DE, USA). To check for purity/contaminants, 16S small subunit RNA genes were amplified with bacterial domain primers 27F and 1492R (Lane, 1991) as described previously (Hamilton *et al.*, 2017). Reactions were performed in triplicate, purified using a QIAquick PCR Purification Kit (Qiagen, Valencia, CA, USA) and sequenced at the Genomics Core Facility of the Huck Institutes of the Life Sciences at the Penn State University. Sequences were assembled and manually checked using using Bio-Edit (v.7.2.5), and checked for chimeras using CHIMERA_CHECK (Cole *et al.*, 2003). Putative chimeras were excluded from subsequent analyses. A single 16S rRNA gene sequence was recovered indicating the culture was pure.

### Genomic sequencing, assembly and completeness

A draft genome of the isolate was generated from genomic DNA extracted as described above. Purified DNA was sequenced with on an Ion Torrent Personal Genome Machine according to the Ion Torrent protocol at the Penn State University sequencing facility. Specific details are provided in the Supplementary Online Material (SOM). The resulting reads were assembled with Newbler assembler version 2.6 (Roche Diagnostics, Basel, Switzerland) resulting in 77 contiguous reads (contigs) of at least 500 bp with an average read depth of ~100 ×. Contigs were annotated using RAST (http://rast.nmpdr.org; Aziz *et al.*, 2008) and manually curated. Genome completeness was evaluated using conserved housekeeping genes (Supplemetary Table S1) and phylogenetic marker genes identified with Phyla-AMPHORA (Wang and Wu, 2013). The draft genome of the LSS cyanobacterium (scaffolds larger than 1000 bp in length) was submitted to the Integrated Microbial Genomes Expert Review automated pipeline from Joint Genomes Institute for annotation of genes and pathways (IMG accession number: 2708742396). In addition, raw reads and assembled scaffolds have been submitted to NCBI (project: PRJNA355315).

### Phylogenetic analysis

The 16S rRNA gene sequence from the LSS isolate was compared to full-length or near full-length sequences in public databases using BLASTN (Altschul *et al.*, 1997). Sequences were added to an existing 16S rRNA alignment in ARB (Ludwig *et al.*, 2004), and manually refined. Maximum likelihood trees were constructed using PhyML (Guindon *et al.*, 2010) with 1000 bootstrap replicates using the general time-reversible model and substitution parameters estimated from the data. The resulting trees were viewed and edited using iTOL (http://itol.embl.de/)(Letunic and Bork, 2016).

For gene-specific phylogenetic analyses, full-length sequences were identified in the genome using functional annotation and BLASTX, translated and verified by BLASTP. Reference datasets were populated by detecting homologs in IMG genomic databases by BLASTP (Altschul *et al.*, 1997). Protein sequences were aligned with MUSCLE (Edgar, 2004) and redundancy in the alignments was reduced through the Decrease Redundancy Program (http://web.expasy.org/decrease_redundancy/). Maximum likelihood trees were constructed using PhyML (Guindon *et al.*, 2010) with the LG+gamma model, 4 gamma rate categories, 10 random starting trees, NNI branch swapping and substitution parameters estimated from the data. The resulting trees were viewed and edited using iTOL (http://itol.embl.de/) (Letunic and Bork, 2016).

### Microsensor measurements

Rates of oxygenic and anoxygenic photosynthesis dependent on varying irradiance and H_2_S concentration were determined in biofilms of the isolate using microsensors. Biofilms of the isolate were grown on presterilized glass fiber filters. For the measurements, single filters were transferred into 500 ml custom-made aquarium (Supplementary Figure S1). The aquarium was equipped with a horizontally stretched polyester fibrous web that separated a bottom and a top reservoir filled with B-HEPES media similar to the setup described in Klatt *et al.*, 2015b (Supplementary Figure S1). The filter was placed on top of the web and held in place with pins. When desired, sulfide was added from a neutralized Na_2_S stock solution (pH 7–7.5) to both bottom and top reservoir to ensure that the biofilm had sulfide supplied from both directions. A circular flow of the water column above the biofilm was achieved by streaming N_2_ gas onto the paraffin–oil surface. Illumination in the visible range was provided by a halogen lamp (Schott KL-2500, Mainz, Germany) mounted above the aquarium. The incident irradiance at the surface of the biofilm was determined with a submerged cosine-corrected quantum sensor connected to a LI-250A light meter (both LI-COR Biosciences).

O_2_, pH and H_2_S microsensors with a tip diameter of 10–30 μm and response time of <1 s were built, calibrated and used as described previously (Revsbech, 1989; Jeroschewski *et al.*, 1996; de Beer *et al.*, 1997). The microsensor tips were positioned at the biofilm surface and always separated by <50 μm during simultaneous O_2_, pH and H_2_S measurements. All measurements were performed at room temperature. Volumetric rates of gross oxygenic photosynthesis (GOP) were estimated based on the dynamics of oxygen concentration after a light-dark shift as described previously (Revsbech and Jørgensen, 1986). Analogously, volumetric rates of gross anoxygenic photosynthesis (GAP) were calculated from the dynamics of H_2_S concentration and pH directly after a light-dark transition (i.e., light-driven *S*_tot_ consumption rates) (Klatt *et al.*, 2016a, 2016b).

Before each measurement in the presence of sulfide, GOP as a function of irradiance (9–289 μmol photons m^−2^ s^−1^) was quantified (photosynthesis-over-irradiance-curve (PI-curve)). Irradiance was then adjusted to a specific value chosen from the PI-curve and GOP was determined again, before addition of sulfide. Following injection of sulfide into the water column, the local H_2_S concentration, GOP and GAP in the surface of the biofilm were monitored until complete depletion of sulfide and recovery of GOP. After recovery of GOP, the PSII inhibitor, 3-(3,4-dichlorphenyl)-1,1-dimethylurea (DCMU; Sigma-Aldrich, St Louis, MO, USA) was added to the water column (final concentration: 10 μM) during exposure to light and before the readdition of sulfide. GOP and GAP in the biofilm were again monitored until complete depletion of sulfide. This procedure was repeated at four light intensities (23, 36, 137 and 180 μmol photons m^−2^ s^−1^, *n*=2 for each intensity) on fresh biofilm samples.

To compare GAP and GOP, all reactant consumption/production rates were converted to theoretical electron transport rates as described previously (Klatt *et al.*, 2015a; 2016a). Briefly, assuming zero-valent sulfur is the primary product of cyanobacterial anoxygenic photosynthesis, rates of total sulfide (*S*_tot_=∑(H_2_S,HS^−^,S^2−^)) consumption were multiplied by a factor of 2 to account for two electrons per consumed sulfide that are theoretically transported and used for NADP^+^ reduction. Analogously, the rates of O_2_ production were multiplied by a factor of 4. To allow for the comparison of results obtained in different biofilm samples, we normalized GOP and GAP to GOP_max_, the maximum rate of GOP electron transport, in the respective biofilm sample.

### CO_2_ photoassimilation

The contribution of PSII-independent anoxygenic photoassimilation of CO_2_ with H_2_S as the electron donor was determined in assays containing the PSII inhibitor DCMU. All experiments were carried out at 30 °C in B-HEPES media at pH 7.2. Exponentially growing cells were harvested by centrifugation, resuspended in fresh O_2_-free media and introduced (10-ml suspensions) into O_2_-free 20-ml capped serum vials. Vials were kept in the dark and purged with N_2_ to remove any remaining O_2_. NaH^13^CO_3_ (Cambridge Isotope Laboratories Inc., Andover, MA, USA) was added to each vial at a final concentration of 100 μM. To assess the PSII-independent anoxygenic CO_2_ assimilation, vials (*n*=3 for each treatment) were amended with 100 μM neutralized Na_2_S and 10 μM DCMU (Sigma-Aldrich). To analyze the effects of Na_2_S or DCMU alone on CO_2_ photoassimilation, vials (*n*=3 for each treatment) were amended with 100 μM Na_2_S or 10 μM DCMU. Natural abundance controls (*n*=3 vials) received unlabeled NaHCO_3_ (100 μM). Vials were incubated at a light intensity of 100 μmol photons m^−2^ s^−1^ under cool white fluorescent lamps. Following incubation, cells were harvested by centrifugation and freeze-dried. Dried samples were treated with concentrated HCl (1 M) to remove excess carbonate, washed with deionized H_2_O to remove excess acid and freeze-dried again. Samples were weighed and placed into tin dishes, sealed and analyzed via an automated elemental analyzer (FlashEA, 1112 series) coupled to a Delta Plus Advantage mass spectrometer (Finnigan DeltaplusXP) (both from Thermo Scientific) as described previosuly (Klatt *et al.*, 2015a). All assays were performed in triplicate.

### Electron transport model

**Figure 1:**
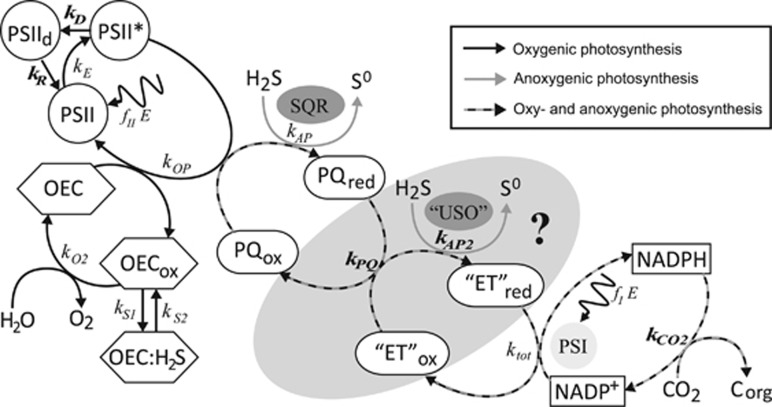
Proposed model for the kinetic control of the redox reactions involved in oxygenic and anoxygenic photosynthesis by strain hensonii. Gray arrows represent reactions involved only in anoxygenic photosynthesis; black arrows are those reactions that are involved only in oxygenic photosynthesis. *E* is the photon flux; PSII, PSII* and PSIId are photosystem II in the ground, excited and degraded/inactive state, respectively; OEC is the oxygen evolving complex. OEC_ox_ is an intermediate formed during H_2_O oxidation that is inhibited by H_2_S. OEC:H_2_S is the inhibited form of this intermediate; SQR is the sulfide quinone oxidoreductase that couples the oxidation of sulfide to the reduction of the oxidized part of the plastoquinone pool (PQ_ox_), which yields zero-valent sulfur and reduced PQ (PQ_red_). ‘ET’ is representative of any intermediate electron transport chain component between PQ and photosystem I (PSI) that serves as the electron acceptor for an unidentified sulfide oxidase (‘USO’). ‘ET’ could be cyt *b*_6_*f*, plastocyanin or cytochrome *c*_553_. The PSI reaction center can receive electrons from the reduced intermediate ‘ET’. In the alternative version of the model that does not involve ‘USO’, PQ directly reduces PSI. These electrons are used to reduce NADP^+^ to NADPH, which serves as the electron donor during CO_2_ fixation. Definitions of the process rates (*k*_*i*_) are given in Table 2. Rates that were introduced to specifically explain the photosynthetic activity patterns in strain hensonii and that are not based on previously described models (Klatt *et al.*, 2015a, 2015b) are highlighted in bold (*k*_D_, *k*_R_, *k*_PQ_, *k*_AP2_, 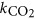). Details of the model are provided in the SOM.

We combined previously described models of (i) the main electron transport chain components in photosynthetically versatile Cyanobacteria (Klatt *et al.*, 2015a) (Figure 1) and (ii) the inhibition kinetics of the oxygen evolving complex (OEC) by H_2_S (Klatt *et al.*, 2015b) (Figure 1) to fit our experimental data of GOP and GAP. Briefly, the model of the oxygenic and anoxygenic electron transport chain focuses on the PQ where both pathways intersect (Figure 1). In this model the affinity of PSII components and SQR towards PQ, as well as light harvested in both PSI and PSII, represent the main regulatory parameters of photosynthetic activity. Inhibitory effects of H_2_S on oxygenic photosynthesis were not considered in this model because such effects were not observed in the specific study organism of Klatt *et al.*, 2015a. To account for inhibitory effects of H_2_S on activity of the LSS cyanobacterium, we extended the model by introducing the interference of H_2_S with the water splitting reaction proposed by Klatt *et al.* (2015b). Briefly, according to this model H_2_S binds to an intermediate of the OEC that is formed at a rate that is determined by the light intensity (Figure 1).

Implementation into a numerical model using the deSolve package in R (Soetaert *et al.*, 2010; http://cran.r-project.org) allowed us to test hypotheses about the regulation of photosynthesis and to fit the experimental data. We introduced additional pathways (rates highlighted in bold in Figure 1) whenever the observed rates could not be explained based on the previously existing models. The fundamental concepts of the models described previously, such as a constant total PQ pool with variable oxidized and reduced fraction (Klatt *et al.*, 2015a), were maintained in the implementation process. Details of the formulation of process rate laws are provided in the SOM.

## Results and discussion

### Isolation of *Leptolyngbya* sp. strain hensonii

Samples of red pinnacle mat collected from the sediment–water interface in LSS were used for isolation by serial dilution. Multiple transfers of the red filaments making up the majority of the mat biomass resulted in an axenic culture of a cyanobacterium which contains chlorophyll *a*, phycocyanin, phycoerythrin and allophycocyanin. The isolate is red colored, filamentous (Supplementary Figure S2), motile and forms pinnacles in pure culture.

The 16S rRNA gene sequence of the isolate is identical to a sequence recovered from the red pinnacle mat in LSS (KP728185; Hamilton *et al.*, 2017). Based on BLASTN analyses against all non-redundant nucleotide sequences in the NCBI-NT database, the isolate is closely related to an uncultured clone from copper mine water (KF287742; 95% sequence identity) and copper mine tailings (JQ769661; 95% sequence identity). The 16S rRNA gene sequence of the isolate also shared 94% sequence identity with clones recovered from the benthic zone of an east Antarctic Lake (DQ181675, DQ181685).

**Figure 2:**
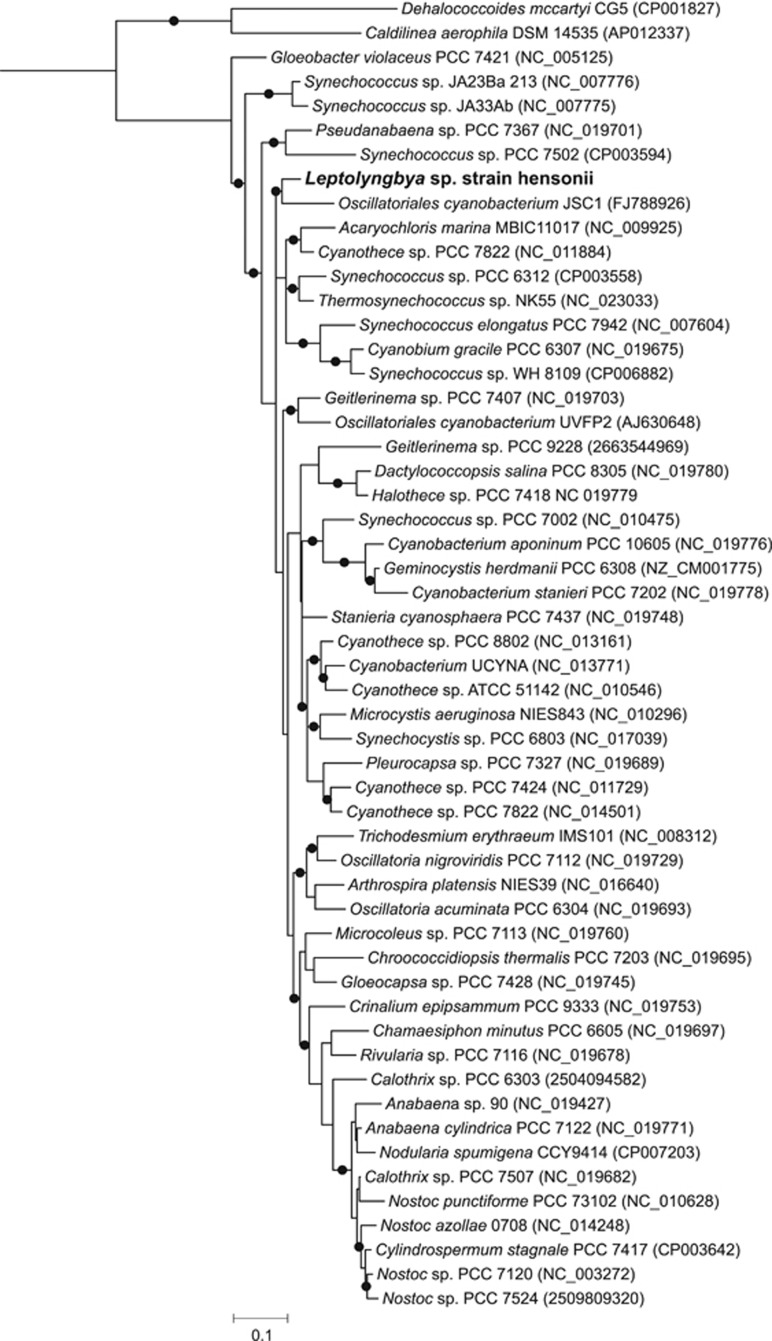
Maximum likelihood phylogenetic 16S rRNA gene tree of closely related Cyanobacteria and *Leptolyngbya* sp. strain hensonii. Accession numbers are provided in parentheses. Circles represent bootstrap support values >85 based on 1000 bootstrap samplings.

The phylogenetic position of the isolate was evaluated by comparing a 1293 bp fragment of the 16S rRNA gene sequence with closely related Cyanobacteria for which draft or full genomes are available (Figure 2). In this analysis, the 16S rRNA sequence of the isolate formed a monophyletic branch (bootstrap value 0.91) with the most closely related sequence—*Leptolyngbya* sp. strain JSC1 within subsection III. *Leptolyngbya* sp. strain JSC1 was isolated from a ferrous iron-rich hot spring with circumneutral pH in Yellowstone National Park (Brown *et al.*, 2010). The LSS isolate is also closely related to *Geitlerinema* sp. PCC 7407 and *Oscillatoriales cyanobacterium* UVFP2 (~94% sequence identity)(Figure 2). A highly enriched culture of *Oscillatoriales cyanobacterium* UVFP2 was obtained from Fuente Podrida, a sulfide-rich spring close to the Cabriel River in Valencia, Spain (Camacho *et al.*, 2005), while the isolation source of *Geitlerinema* sp. PCC 7407 has not been published. The pinnacle-forming LSS cyanobacterium strain was named *Leptolyngbya* sp. strain hensonii, for its growth habit resembling the fur of Jim Henson’s famous puppets. We acknowledge that *Leptolyngbya* are polyphyletic; however, a systematic nomenclature for Cyanobacteria has not been published (Komárek, 2016) and the description of *Leptolyngbya* is consistent with our isolate—long filaments with solitary or coiled clusters and fine mats.

### Genome features

The draft genome of strain hensonii contains 77 contigs and 5 940 030 bp with an average GC content of 52.3% (Table 1). The draft genome encodes 61 tRNAs and 5627 protein coding genes (Table 1), including all of the conserved housekeeping genes (Supplementary Table S1) and 96% of the phylum-specific marker genes identified with Phyla-AMPHORA. All of these metrics suggest that the genome is nearly complete.

The genome encodes the enzymes necessary for aerobic photoautotrophic growth including Form I RuBisCO and a complete Calvin–Benson cycle; two high affinity terminal oxidases, a cytochrome *c* oxidase and a *bd*-type quinol oxidase; PSI and PSII; chlorophyll biosynthesis pathway enzymes; and a cytochrome *b*_6_*f* complex. The *bd*-type quinol oxidase and cytochrome *c* oxidase differ in their affinity for oxygen (0.35 vs 1.0 μM) (Pils and Schmetter, 2001), and the former is postulated to be expressed under low oxygen conditions (Hart *et al.*, 2005). Genes encoding a succinate dehydrogenase (*sdhABC*), an F-type ATPase and a NAD(P)H:quinone oxidoreductase (NDH) are also present. Thus, hensonii is expected to be capable of aerobic respiration under variable O_2_ concentrations. In the environment hensonii is indeed exposed to fluctuating O_2_ over a diel cycle (de Beer *et al.*, 1997). O_2_ is, however, not available from the water column *in situ* but is exclusively produced by oxygenic photosynthesis, with hensonii as a main source. Under *in situ* conditions in LSS the enzymatic machinery for aerobic respiration likely serves to maintain cellular redox balance, with the terminal oxidases, for instance, serving as electron valves for the photosynthetic electron transport reactions (Supplementary Figure S3). This implies that terminal oxidases would thus never be used in the presence of the inhibitory H_2_S (Beauchamp *et al.*, 1984; Cooper and Brown, 2008). The genome also encodes the enzymatic machinery necessary for assimilatory nitrate reduction and assimilatory sulfate reduction and nitrogen fixation via a Mo-dependent nitrogenase.

Three enzymes integral to photosynthesis—coproporphyrinogen III oxidase, heme oxygenase and Mg-protoporphyrin IX monomethylester cyclase—require oxygen for activity. However, oxygen levels at the water depth hosting the red pinnacle mats in LSS reach only 0.2 μM oxygen (de Beer *et al.*, 2017). Genes encoding alternative forms of these enzymes have been observed in genomes of Cyanobacteria from diverse environments (Panek and O’Brian, 2002) and, in the cyanobacterium *Synechocystis* sp. PCC 6803, the alternative forms of these enzymes are expressed under low oxygen conditions (Aoki *et al.*, 2011). Consistent with environmental conditions in the natural habitat of the isolate, we found homologs of both the aerobic and anaerobic forms of these enzymes in the strain hensonii genome. Multiple *psbA* genes (which encode a subunit of PSII) have been observed in the genomes of sulfide-tolerant and/or sulfide-using Cyanobacteria (Grim and Dick, 2016). These additional copies of *psbA* facilitate oxygenic photosynthesis under conditions of varying oxygen and light (Mohamed *et al.*, 1993). In the strain hensonii draft genome, we observed the copies of the canonical oxygenic group 4 *psbA* (Cardona *et al.*, 2015) as well as a group 3 *psbA*, which have been recovered from cyanobacterial genomic bins from a low-oxygen cyanobacterial mat in the Middle Island Sinkhole (Voorhies *et al.*, 2012), and a group 2 *psbA.* Transcripts of group 2 *psbA* have been observed under microaerobic conditions in cultures of *Synechocystis* PCC 6803, *Anabaena* PCC 7120 and *Thermosynechococcus elongatus* (Sicora *et al.*, 2009).

The draft genome of *Leptolyngbya* sp. strain hensonii encodes a single SQR—specifically an SQR type F. SQR catalyzes the oxidation of sulfide to zero-valent sulfur and may have a physiological role in both energy transduction and sulfide detoxification. SQR has also been implicated in anoyxgenic photosynthetic activity in Cyanobacteria (Shahak *et al.*, 1998) (see Figure 1). SQR sequences can be divided into seven classes (A, B, C, D, E, F or X) and a single genome can encode multiple SQR homologs (Gregersen *et al.*, 2011). SQRA are typically found in Cyanobacteria, Proteobacteria and Aquificaceae. SQRD and SQRX form two paralogous clades—SQRD homologs are encoded by strains of GSB, Proteobacteria and Actinobacteria (Gregerson *et al.*, 2011), while SQRX homologs are encoded by GSB. No representative of SQRC has been demonstrated to oxidize sulfide. SQRB homologs are often recovered from eukaryotes, while SQRE catalyzes sulfide oxidation in the archaeon *Acidianus ambivalens* (Brito *et al.*, 2009). SQRF homologs are commonly observed in the genomes of in GSB, Proteobacteria, Aquificaceae and Cyanobacteria (Supplementary Figure S5); however, an SQRF from a Cyanobacteria has not been characterized. In the GSB *Chlorobaculum tepidum*, SQRF is important for growth at high sulfide concentration (≥4 mM) (Chan *et al.*, 2009; Holkenbrink *et al.*, 2011). The hensonii SQRF is only distantly related (25% sequence identity) to the Type A SQR sequences of *Aphanothece halophytica* and *Geitlerinema* sp. PCC 9228 (formerly *Oscillatoria limnetica*), which have been implicated in anoxygenic photosynthesis in these isolates (Cohen *et al.*, 1986).

Red pinnacle mats collected from LSS contain elevated concentrations of hopanoids, including those methylated at the C-2 position (Hamilton *et al.*, 2017). The isolate genome encodes homologs of two enzymes that are presumably necessary for the biosynthesis of 2-methylhopanoids—a squalene–hopene cyclase and a radical SAM methylase (HpnP) (Welander *et al.*, 2010). The translated HpnP homolog branches with other cyanobacterial HpnP sequences (Supplementary Figure S6) and is identical to the *hpnP* transcript recovered from LSS (Hamilton *et al.*, 2017). These results suggest that the isolate is a source of 2-methyl hopanoids in LSS. Several lines of evidence suggest that anoxic conditions favor the production of 2-methyl hopanoids: (1) an increased abundance in Proterozoic rocks compared to Phanerozoic rocks (Summons *et al.*, 1999); (2) increased abundance in rocks recording oceanic anoxic events in the Phanerozoic (Knoll *et al.*, 2007; Talbot *et al.*, 2008; Cao *et al.*, 2009; Kasprak *et al.*, 2015); and (3) higher abundance of *hpnP* genes in environments where anoxic conditions prevail (Ricci *et al.*, 2013). The recovery of 2-methyl hopanoids from anoxic mats in LSS is consistent with these lines of evidence, and the isolation of a cyanobacterium with the genetic machinery to synthesize 2-methyl hopanoids will facilitate future studies aimed at determining their functions in adaptation and/or metabolism.

### Strain hensonii performs anoxygenic photosynthesis

**Figure 3:**
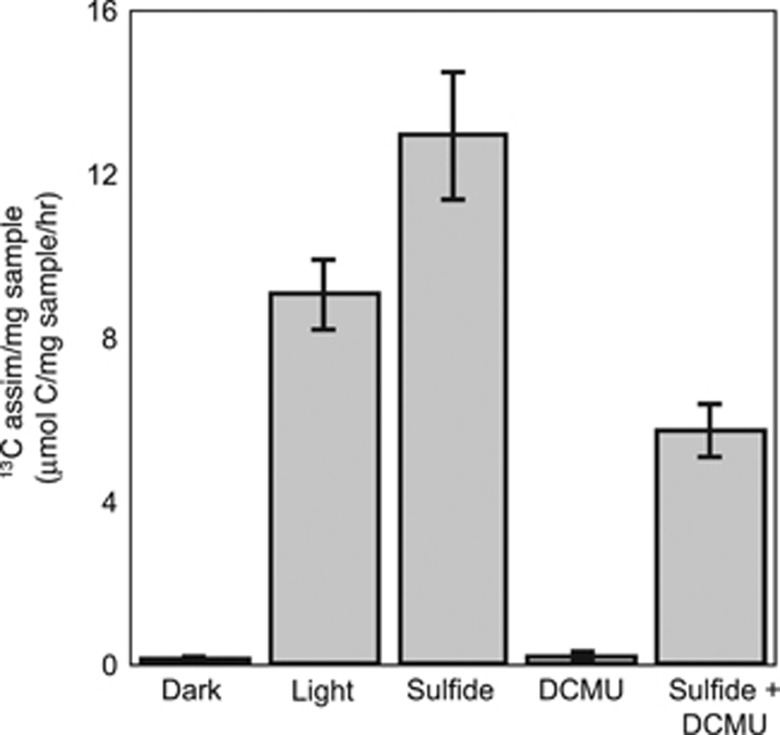
Inorganic carbon assimilation by *Leptolyngbya* sp. strain hensonii. Error bars obtained from triplicate measurements.

**Figure 4:**
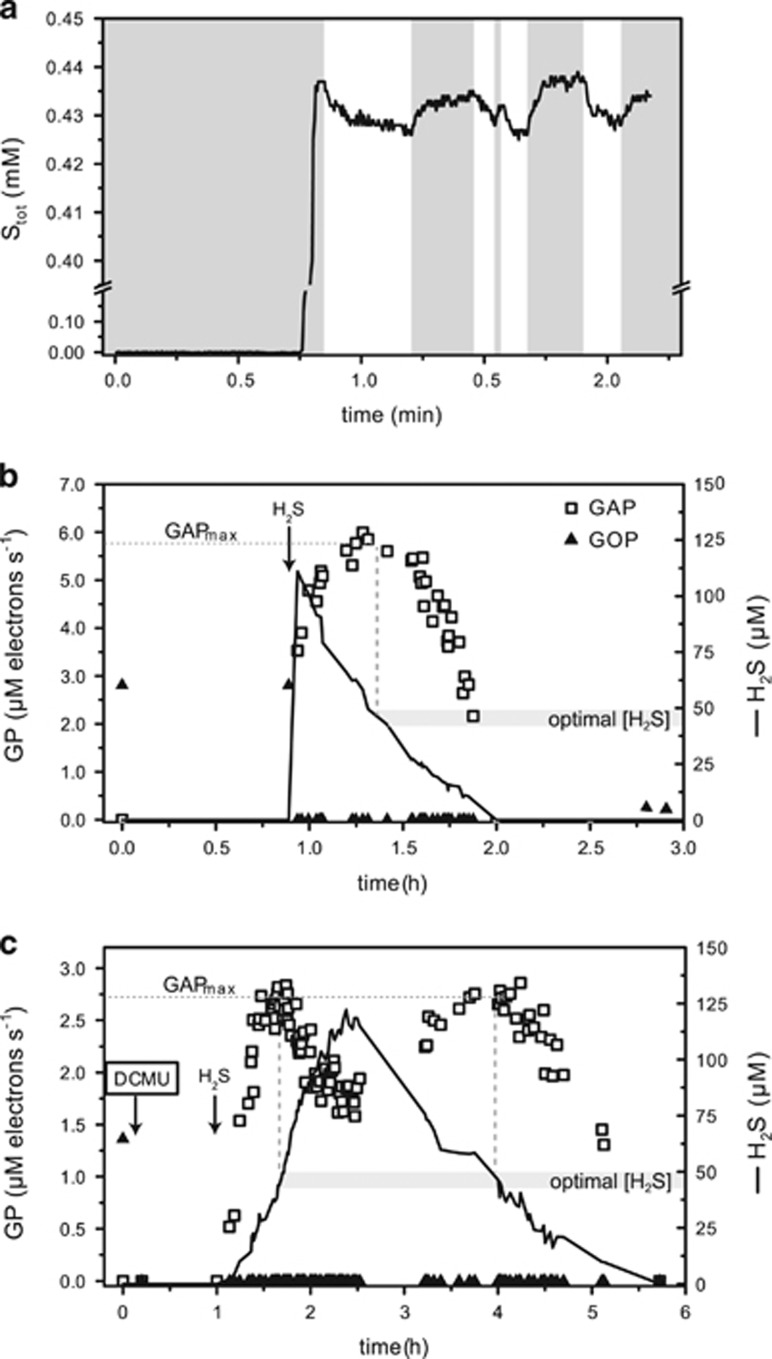
Example of the light-induced dynamics of total sulfide concentration (**a**) based on which volumetric GOP and GAP (**b** and **c**) were calculated. In (**a**) dark intervals are indicated by the dark shaded area. All measurements were performed in the uppermost layer of three different strain hensonii samples in the absence (**a** and **b**) and presence (**c**) of DCMU. In the specific example shown in (**a**), sulfide was injected in the dark. Injections in (**b** and **c**) were done in the light (137 μmol photons m^−2^ s^−1^ incident irradiance) after determination of GOP in the absence of H_2_S. After injection (indicated by the arrows), GAP was monitored. The corresponding local H_2_S concentration over time is also shown. As the neutralized Na_2_S solution was injected at variable distance from the biofilm, H_2_S concentration in the biofilm increased at different rates. The decrease of H_2_S concentration was caused by a combination of outgassing, local pH modulation by photosynthesis in the biofilm and photosynthetic consumption of sulfide. In (**b**) inhibitory concentrations of H_2_S were reached immediately after injection of H_2_S close to the biofilm. In (**c**) rates of GAP increased slowly with H_2_S concentration until GAP_max_ was reached. H_2_S concentrations >~44 μM led to inhibition of GAP (compare to Figure 5). Upon decrease of H_2_S, rates of GAP instantaneously recovered and only decreased again upon H_2_S limitation. All rates were converted to photosynthetic electron transport rates.

Because the *Leptolyngbya* sp. strain hensonii lives under anoxic and sulfidic conditions *in situ* (de Beer *et al.*, 2017) and the genome encodes at least one SQR protein, we hypothesized that the isolate could perform anoxygenic photosynthesis. Indeed, in the presence of sulfide and the PSII inhibitor DCMU, the isolate assimilated 5.7 (±0.71) μmol C mg dry weight^−1^ suggesting anoxgenic photosynthetic activity (Figure 3). The capability to perform anoxygenic photosynthesis was confirmed by microsensor-based measurements in the absence and presence of DCMU (Figure 4). In fact, microsensor-based measurements indicate that no induction time is necessary for anoxygenic photosynthesis (Figure 4), even if strain hensonii had been grown aerobically before exposure to sulfide. This is in contrast to other characterized Cyanobacteria capable of performing anoxygenic photosynthesis that require ~2 h in the presence of sulfide before performing this activity (Oren and Paden, 1978; Cohen *et al.*, 1986; Klatt *et al.*, 2015a). Oxygenic and anoxygenic photosynthesis were never observed to occur simultaneously during our experiments because oxygenic photosynthesis was inhibited by H_2_S concentrations of 1 μM or lower based on the detection limit of the specific H_2_S sensor used (Figure 4).

### Regulation of anoxygenic photosynthesis

In addition to oxygenic photosynthesis, several Cyanobacteria are capable of using sulfide as an electron donor, that is, performing anoxygenic photosynthesis using only PSI (Cohen *et al.*, 1975a, 1975b; de Wit and van Gemerden, 1987); however, the mechanism for regulating anoxygenic photosynthetic activity is different between isolates (Cohen *et al.*, 1986; Garcia-Pichel and Castenholz, 1990; Klatt *et al.*, 2015a; 2016a). Below we report specific activity patterns dependent on H_2_S concentration and irradiance of strain hensonii in laboratory experiments and discuss plausible regulation mechanisms (see the SOM for additional details and discussion).

### Light and H_2_S concentration

**Figure 5:**
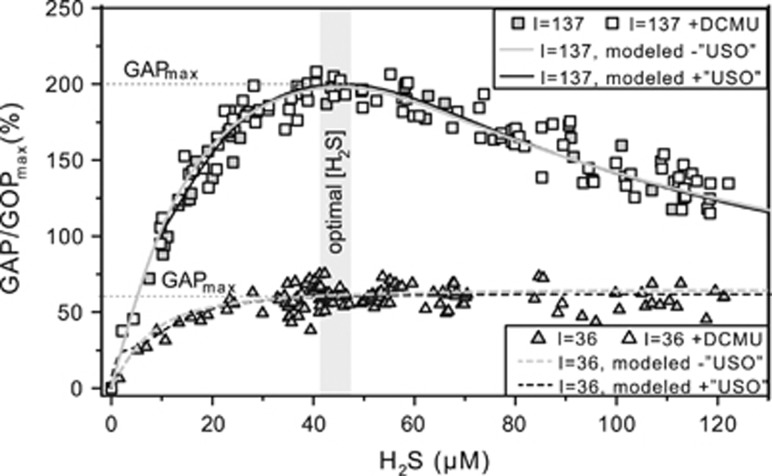
Volumetric gross rates of anoxygenic photosynthesis dependent on H_2_S concentration measured at 36 and 137 μmol photons m^−2^ s^−1^ in the absence and presence of DCMU. The values are normalized to the maximum electron transport rate of oxygenic photosynthesis at the optimal irradiance 137 μmol photons m^−2^ s^−1^ (GOP_max_, measured before the injection of sulfide; Figure 4). The dotted horizontal gray lines indicate the light-dependent maximum rate of GAP at optimal H_2_S concentration (GAP_max_). The solid and dashed gray lines represent the output of the model that does not consider the presence of an ‘USO’, but light-dependent changes of SQR activity as described by Figure 1 and in Table 2. The black lines are the output of the model built on the assumption of an ‘USO’.

Anoxygenic photosynthesis was regulated by both irradiance and H_2_S concentration—GAP increased with increasing H_2_S concentration until a light-dependent maximum (GAP_max_; dashed horizontal lines in Figure 5) was reached. The initial increase of GAP over low H_2_S concentrations and the saturation effect at higher concentrations resembled H_2_S-dependent Michaelis–Menten kinetics. The initial slope of the increase and the maximum GAP were also light-dependent. All patterns were strictly dependent on H_2_S concentration and were not affected by the temporal dynamics of exposure to sulfide (see H_2_S dynamics in Figures 4b and c). As expected, the inhibition of GOP by DCMU did not have an effect on GAP (compare open and closed symbols in Figure 5) because GOP was also inhibited by H_2_S.

### H_2_S dependency suggests kinetic regulation of GAP

The increase of GAP with H_2_S until a light-dependent maximum (GAP_max_; Figure 5) is consistent with previous observations (Cohen *et al.*, 1986; Garcia-Pichel and Castenholz, 1990; Klatt *et al.*, 2015a, 2016a) and can be explained using a previously described model of the anoxygenic photosynthetic electron transport reactions (Klatt *et al.*, 2015a). According to the model, the H_2_S oxidation rate by SQR is concentration-dependent and SQR donates electrons to the PQ. Reoxidation of PQ is governed by light harvested in PSI. Thus, H_2_S oxidation proceeds at a rate that depends on the affinity of SQR for H_2_S and the oxidized part of the PQ pool (*k*_AP_; Figure 1; Table 2; SOM) and is consequently governed by H_2_S concentration and the availability of oxidized PQ.

#### Light dependency suggests multiple sulfide-oxidizing enzymes

Irradiance had two effects on GAP: (a) it determined GAP_max_ and (b) it affected the initial slope of GAP increase with H_2_S concentration (Figure 5). The first effect can be explained by considering that rates of H_2_S oxidation can only increase with H_2_S concentration until PSI becomes a bottleneck for electron transport reactions (Klatt *et al.*, 2015a). Specifically, the light energy harvested in PSI dictates the maximum electron transport rate in the irradiance range below light saturation (*k*_tot_; Figure 1; Table 2; SOM), which also represents GAP_max_ (Figure 5).

Intriguingly, the light-dependent slope of the increase in GAP with H_2_S concentration could not be explained using the previously described model for anoxygenic photosynthesis in Cyanobacteria. Different light-dependent slopes of GAP have been observed in a cyanobacterium, but these could be explained by GAP and GOP occurring simultaneously, with the two photosynthetic modes competing for the PQ pool (Klatt *et al.*, 2016a). However, GAP and GOP are not performed concurrently in strain hensonii, suggesting light must have more complex, previously unconsidered, effects on GAP.

To fit our data with the model of the anoxygenic photosynthetic electron transport reactions, we had to suspend a basic assumption: a steady pool of a single sulfide-oxidizing enzyme. Specifically, we had to make one of two plausible assumptions instead: (a) The isolate is equipped with one or multiple types of SQR and the abundance of these enzymes is dependent on irradiance. For instance, if, at higher light intensities the synthesis of SQR is upregulated and thus has a higher *v*_max_ (see *k*_AP_B_ in Table 2; SOM), the result would be more active SQRs and an increased maximum rate of H_2_S oxidation, manifested in a steeper initial slope in GAP (gray lines in Figure 5 for model output). (b) There are two types of sulfide-oxidizing enzymes (SQR and unidentified sulfide oxidase ‘USO’ in Figure 1 and Supplementary Figure S4) with different affinities for H_2_S, with SQR donating electrons to the PQ pool and ‘USO’ donating electrons into the electron transport chain at some other level, most likely to cytochrome *b*_*6*_*f* or plastocyanin (or cytochrome *c*_553_), which are encoded in the genome of hensonii (see ‘USO’ and *k*_AP2_ in Figure 1 and Table 2; black lines in Figure 5 and Supplementary Figure S4 for model output; and SOM for more details). Although the genome is estimated to be 96% complete, we cannot rule out the possibility that the isolate encodes a second SQR. We still consider hypothesis (a) unlikely because, to the best of our knowledge, the regulation of SQR activity and/or abundance by light has not been observed in other phototrophs. Assumption (b) on the other hand eliminates the need for a direct effect of irradiance on enzyme activity or regulation of transcription to explain the light and H_2_S dependency of GAP. Other sulfur-oxidizing enzymes including flavotocytochrome *c*, dissimilatory sulfur reductase or sulfite:cytochrome *c* oxidoreductase were not encoded by the draft genome. Still, multiple studies point towards enzymes other than SQR involved in cyanobacterial AP. Namely, pure cultures of *Phormidiaceae cyanobacterium* SAG 31.92 (formerly *Microcoleus chthonoplastes* strain 11) oxidize sulfide to thiosulfate (de Wit and van Gemerden, 1987), while other anoxygenic Cyanobacteria (i.e., *Oscillatoria* spp.) oxidize sulfide to elemental sulfur that accumulates extracellularly (Cohen *et al.*, 1975a, 1975b; Castenholz and Utkilen, 1984) or to or sulfite (Rabenstein *et al.*, 1995). For now, the second sulfide-oxidizing enzyme, however, remains hypothetical. Clearly, both hypotheses (a) and (b) invite future testing.

Independent of whether using assumption (a) or (b) outlined above, we consistently obtained the best fit for our experimental data (lines in Figure 5 and Supplementary Fig S4) by assuming that the *Leptolyngbya* sp. strain hensonii’s SQR for H_2_S with apparent *K*_M_ values between 0.05 and 0.2 mM (see SOM for details). SQRF from the GSB *Chlorobaculum tepidum* was found to have *K*_M_ values in the millimolar range (Chan *et al.*, 2009). Conversely, the well-characterized SQR of *Geitlerinema* sp. PCC 9228 (type A SQR), which has been implicated in anoxygenic photosynthesis, has a high affinity for sulfide (Oren and Paden, 1978). Regardless, the *K*_M_ of SQRs for sulfide can vary substantially.

### Effects of H_2_S on oxygenic photosynthesis

**Figure 6:**
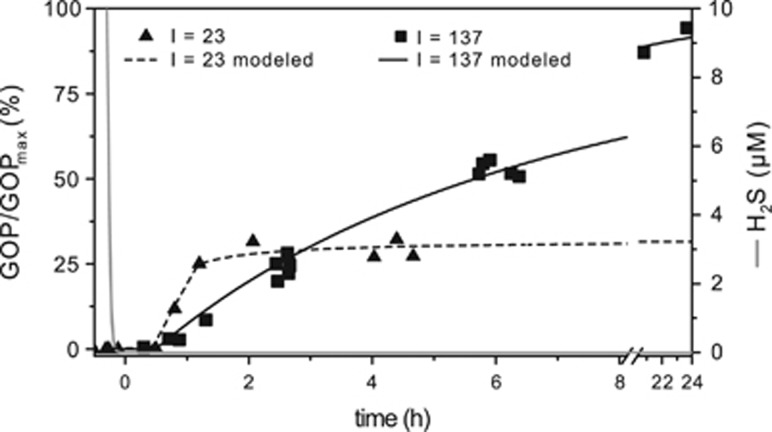
Recovery of the volumetric GOP after the depletion of H_2_S by strain hensonii (at time point 0 h) during exposure to 23 (triangles) and 137 (squares)  μmol photons m^−2^ s^−1^. The black lines represent the output of the simulation of the experimental data based on the model described in Figure 1 and in Table 2.

Both metabolically versatile and obligate oxygenic Cyanobacteria inhabit microbial mats characterized by fluctuating redox conditions and intermittent exposure to H_2_S. Diverse metabolic processes in Cyanobacteria can be affected by H_2_S—most prominently, sulfide can inhibit oxygenic photosynthesis (Oren *et al.*, 1979) by poisoning PSII. In strain hensonii, GOP was instantaneously inhibited by H_2_S (Figure 4c). Oxygenic photosynthesis did not recover until H_2_S concentrations remained at ~0 μM for ~30 min (Figure 6) and the rate of GOP recovery decreased with increasing light intensity.

The mechanism of inhibition is thought to be the interaction of H_2_S with the OEC in PSII (Cohen *et al.*, 1986; Garcia-Pichel and Castenholz, 1990). To test if the kinetics of the OEC inhibition mechanism can explain the delayed recovery of oxygenic photosynthesis, we used a previously described model of OEC inhibition (Klatt *et al.*, 2015b). We found that the light-independent ~30 min delay of GOP recovery in strain hensonii can be understood by assuming that H_2_S only slowly dissociated from the OEC even after external H_2_S was depleted—that is, the back reaction to an active non-inhibited OEC (*k*_S2_ in Figure 1 and Table 2) is slow. As soon as non-inhibited OEC is available, oxygenic photosynthesis can resume.

To explore the light dependency of the recovery rate of GOP, we introduced degradation and repair rates of PSII (D_1_ subunit) into the model (*k*_D_ and *k*_R_, respectively, in Figure 1; Table 2; see SOM for more details). We assumed that the rate of degradation is dependent on light intensity and the level of OEC inhibition. This is because excitation energy harvested in PSII cannot be used efficiently for photochemical reactions if a part of the OEC pool is inhibited. The ‘unused’ fraction of energy is expected to enhance degradation. In other words, H_2_S inhibition of the OEC enhances photoinhibition. Upon reinstatement of the complete pool of uninhibited OEC, light intensity becomes the only factor controlling recovery of GOP. If the light intensity is high, the rate of PSII degradation will still substantially exceed the rate of PSII repair, causing slow recovery. In contrast, low light intensities will allow for a rapid decrease in photoinhibition rates and consequently a fast recovery of oxygenic photosynthesis.

The assumptions that (i) the 30-min delay in recovery is caused by OEC inhibition kinetics and (ii) the recovery rate of GOP depends on OEC inhibition are not independent—both are caused by an interplay between the kinetics of OEC inhibition and photoinhibition reactions based on the model depicted in Figure 1 (and described in the SOM). The results of the implementation of this concept into the numerical model are in remarkable agreement with the experimental data (lines in Figure 5). Thus, we propose that GOP inhibition is solely controlled by inhibition kinetics and does not invoke additional regulatory mechanisms, such as H_2_S-driven degradation of PSII and a delayed resynthesis of PSII. Still, future studies of this physiology including transcriptomic studies are necessary to fully elucidate the mechanism of GOP inhibition.

### Effect of H_2_S on reactions downstream of PSI

Besides the direct regulatory effects on the initial oxidation reactions of oxygenic and anoxygenic photosynthesis, our data suggest that sulfide also affects reactions downstream of PSI, likely reactions of the Calvin cycle. H_2_S appears to both enhance and inhibit these reactions, with the balance between these contrasting effects depending on light and H_2_S conditions.

Inhibition of anoxygenic photosynthesis at non-optimal H_2_S concentrations was previously observed by Cohen *et al.* (1986). Intriguingly, in *Leptolyngbya* sp. strain hensonii, the inhibition was light-dependent. During exposure to the optimal light intensity for GOP (137 μmol photons m^−2^ s^−1^), GAP_max_ was reached at ~44 μM H_2_S, followed by a pronounced decrease in GAP with increasing H_2_S concentration (Figure 5). The pronounced decrease of GAP was, however, not observed during exposure to lower light intensities (Figure 5). Because light has an effect on this inhibition, a simple substrate inhibition of SQR cannot account for the decrease in GAP. Using our model, we found that light-dependent inhibition by H_2_S can best be explained by assuming that H_2_S inhibits a reaction downstream of PSI (e.g., 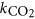 in Figure 1 and Table 2, see lines in Figure 5 for model output and SOM for more details), which only has a role when the maximum GAP is not exclusively controlled by irradiance, that is, at light intensities where the rate of CO_2_ fixation limits the overall electron transport rate.

**Figure 7:**
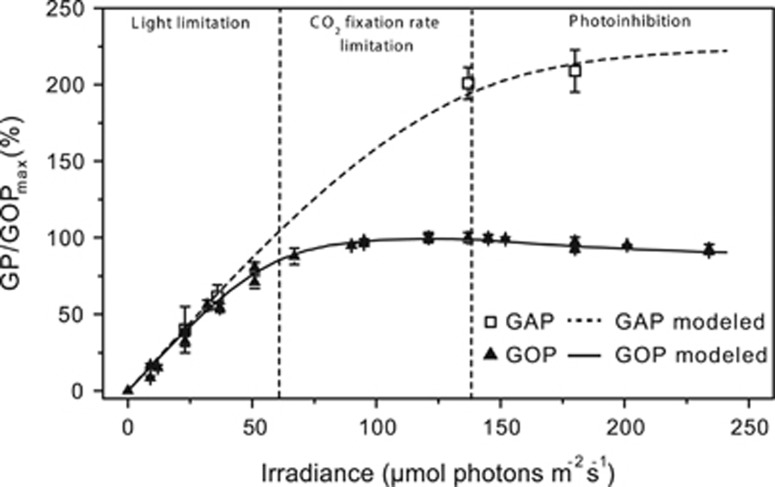
Volumetric GOP with lines representing the results of the model described by Figure 1 and in Table 2. The resultant photosynthesis-over-irradiance-curves (PI-curve) for GOP is divided into operational irradiance ranges based on the plausible rate-limiting steps of oxygenic photosynthetic electron transport. The experimental data were fitted with the model of Eilers and Peeters (1988) for PI-curves by nonlinear regression (data not shown) to determine the optimal light intensity (137 μmol photons m^−2^ s^−1^) and the biomass-dependent GOP at this optimal light intensity (GOP_max_). Normalization of all rates from each separate measurement to GOP_max_ revealed that the activity relative to GOP_max_ was highly reproducible and independent of the biomass in the surface layer. For GOP, the average of replicate measurements in each biofilm sample is shown (*n*=3–7; error bars are standard deviation). Values for GAP shown here are the average maximum rates at optimal H_2_S concentration (GAP_max_, see Figure 5) in the presence and absence of DCMU (*n*=4).

The enhancement of reaction rates downstream of PSI became apparent in the observation that rates of anoxygenic photosynthesis can exceed the rates of oxygenic photosynthesis (Figure 7, note that GAP in Figure 5 is up to 200% of GOP_max_). During exposure to 36 μmol photons m^−2^ s^−1^, GAP did not exceed GOP at any H_2_S concentration (Figure 5 and Figure 7). However, the maximum GAP (in electrons) was roughly two times higher than GOP_max_ during exposure to 137 and 180 μmol photons m^−2^ s^−1^ (Figure 7). An enhancement of photosynthetic rates by sulfide was confirmed by ^13^C-bicarbonate incubations in the absence and presence of DCMU (Figure 3). In the presence of sulfide (without DCMU), *Leptolyngbya* sp. strain hensonii incorporated higher amounts of ^13^C-bicarbonate (12.9 (±1.61)  μmol C assimilated mg dry weight^−1^) compared to cells that received no sulfide (9.07 (±0.92)  μmol C assimilated  mg dry weight^−1^) (Figure 3). The lower assimilation in cells that received both DCMU and sulfide is presumably because DCMU prevented the switch to oxygenic photosynthesis upon depletion of sulfide.

We again used our model to identify the most likely mechanism for the enhancement of GAP. We found the best agreement with our experimental data by assuming that (i) H_2_S upregulates rates downstream of PSI (*γ* in 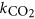 in Table 2 and Figure 1; SOM for more details) and (ii) the increase in electron transport rate is further supported by excitation energy transfer from PSII to PSI, which is regulated by the redox state of the PQ pool (*β* in *k*_tot_ in Table 2; Figure 1; SOM for more details). Thus, H_2_S has no enhancing effect at low light intensities because light harvested in PSI limits electron transport rates (Figure 7). Around the optimal light intensity GOP becomes rate limited by CO_2_ fixation reactions in the Calvin cycle (Sukenik *et al.*, 1987; Cardol *et al.*, 2011) and enhancement and inhibition can take effect (see lines in Figures 5 and 7 for model output).

Based on these data, we propose that H_2_S has two regulatory effects downstream of PSI: It enhances photosynthetic rates at saturating light intensities if concentrations of H_2_S are below 44 μM. Above this threshold, inhibitory effects outweigh the enhancing effect of H_2_S on reactions downstream of PSI. The mechanisms behind enhancement and inhibition warrant further research.

### Summary: complex response based on simple mechanisms

The physiological responses of hensonii to H_2_S are complex: (i) No induction time is necessary for anoxygenic photosynthesis, which suggests that the sulfide-oxidizing machinery is constitutively expressed. (ii) The rates of anoxygenic photosynthesis are regulated by both H_2_S and irradiance. Specifically, rates of anoxygenic photosynthesis increase with H_2_S at a light-dependent slope until light limitation occurs or until inhibitory effects of H_2_S occur, which are more pronounced at higher irradiance. (iii) Under the optimal light conditions, rates of anoxygenic photosynthesis are nearly double that of oxygenic photosynthesis. We suggest that (ii) and (iii) can be explained based on concerted responses of multiple elements involved in oxygenic and anoxygenic photosynthesis: the kinetics of sulfide oxidation by SQR and an ‘USO’, enhanced excitation energy transfer from PSII to PSI upon exposure to sulfide, and enhancing and inhibitory effects of sulfide on reactions downstream of PSI, most likely in the Calvin cycle. (iv) O_2_ production is inhibited by H_2_S concentrations <1 μM and remains inhibited for ~30 min even after depletion of sulfide, wherein the recovery rate of oxygenic photosynthesis after this lag phase is dependent on irradiance. Intriguingly, these observations can be explained by considering the kinetics of OEC inhibition and relaxation, and the kinetics of photoinhibition, that is, PSII/D1 degradation and repair. Therefore, the activity patterns of strain hensonii in response to sulfide and irradiance are thus likely based on relatively simple, instantaneous mechanisms that do not necessarily involve adjustments of the enzyme equipment.

### Ecophysiology of strain hensonii

In pure culture, strain hensonii requires no induction time to perform anoxygenic photosynthesis and consumes sulfide until oxygenic photosynthesis is no longer inhibited. The switch between oxygenic and anoxygenic photosynthesis in strain hensonii is subject to complex regulatory pathways, but essentially depends on light and sulfide. In this respect, our results are consistent with previous characterizations of Cyanobacteria inhabiting sulfidic environments. However, the sluggish recovery (~30-min delay) of oxygenic photosynthesis following depletion of H_2_S is not consistent with the observed success of the isolate *in situ*. Based on the abundance of 16S rRNA gene sequences affiliated with hensonii recovered from LSS mat (Hamilton *et al.*, 2017) and the physiology of the strain described here, it is likely that this organism has a key role in shaping this highly dynamic mat microenvironment.

**Figure 8:**
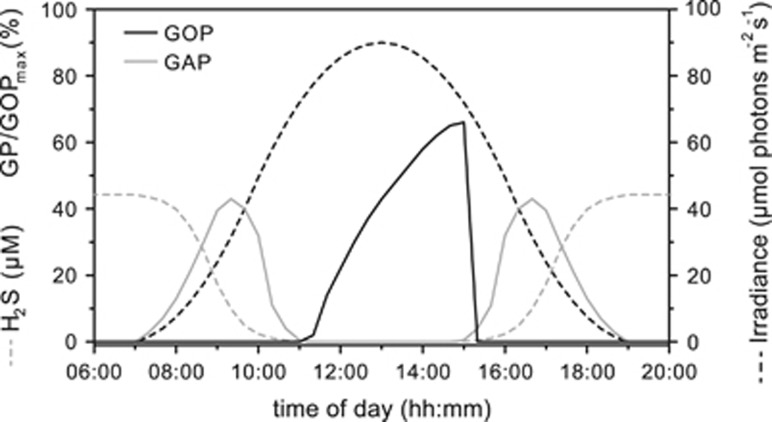
Simulation of irradiance and H_2_S dynamics and the corresponding photosynthetic activity over a diel cycle in the upper layer of a *Leptolyngbya* sp. strain hensonii-dominated microbial mat, using the model described in Figure 1 and Table 2.

*In situ*, the cyanobacterial layer of the LSS mat transitions elegantly between photosynthetic modes over the diel light cycle: In the early morning anoxygenic photosynthesis dominates. In the evening, the cyanobacterial layer transitions back to anoxygenic photosynthesis. To illustrate that the physiology of strain hensonii is not consistent with *in situ* observations, we used our model to simulate the activity of the isolate in LSS over a diel cycle (Figure 8). In our hypothetical biofilm, we assumed that irradiance in the late morning is high enough for complete depletion of H_2_S in the uppermost layers because sulfide supply from underneath is capped by cyanobacterial anoxygenic photosynthesis in deeper layers as has been observed in natural systems including LSS (Klatt *et al.*, 2016b; de Beer *et al.*, 2017). Our model predicts that when sulfide becomes locally depleted in the uppermost layer due to anoxygenic photosynthesis in deeper layers, there is a delay between anoxygenic and oxygenic photosynthetic activity because strain hensonii cannot switch instantaneously from anoxygenic to oxygenic photosynthesis. It seems highly unlikely that a cyanobacterium exhibiting 30 min of photosynthetic inactivity at high light is competitive in the environment and the lag was not observed *in situ* in LSS (de Beer *et al.*, 2017).

To understand how strain hensonii can still be successful in the environment, we need to consider that anoxygenic photosynthesis in the cyanobacterial layer of the LSS mat is fueled by three sources of H_2_S: diffusion from underlying sediment, diffusion from the water column and locally produced H_2_S within the cyanobacterial layer of the mat (de Beer *et al.*, 2017). Locally produced sulfide could cryptically fuel cyanobacterial anoxygenic photosynthesis (de Beer *et al.*, 2017). This means that anoxygenic photosynthesis at high light could be operational even though H_2_S concentrations approach <1 μM—concentrations low enough to allow for oxygenic photosynthesis to start. Thus, strain hensonii could remain photosynthetically active throughout the photoperiod. This implies a very close beneficial interaction with sulfate reducing bacteria. The result is a cyanobacterial dominated mat in a delicately poised environment, the productivity of which is largely controlled by local sulfate reduction.

Microbial mat systems likely represent hotspots of evolution including sulfur cycling processes and photosynthesis (Nisbet and Sleep, 2001). Even the earliest oxygenic phototrophs were likely exposed to intermittently sulfidic conditions in the immediate microenvironment despite largely ferruginous conditions in the oceans during the Archean and much of the Proterozoic (Lyons *et al.*, 2014). Early Cyanobacteria might have had to develop strategies to cope with H_2_S toxicity that have been refined over the following billions of years (Castenholz, 1977; Garlick *et al.*, 1977; Oren *et al.*, 1979; Cohen *et al.*, 1986; Miller and Bebout, 2004). In LSS and in the hensonii isolate, these strategies are not necessary—the cyanobacterial part of the mat performs anoxygenic photosynthesis until enough sulfide is consumed to enable oxygenic photosynthesis, whereas the Chlorobi-dominated deeper mat continuously performs anoxygenic photosynthesis due to sulfide production from locally closely associated sulfate reducing organisms. The physiology of the hensonii strain is consistent with ecological success in this environment: (i) no induction time is necessary for anoxygenic photosynthesis; (ii) rates of anoxygenic photosynthesis are regulated by both H_2_S and irradiance; (iii) O_2_ production is inhibited by H_2_S concentrations as low as 1 μM and the recovery rate of oxygenic photosynthesis is dependent on irradiance; (iv) rates of anoxygenic photosynthesis can be nearly double those of oxygenic photosynthesis. While the evolutionary history of metabolically versatile Cyanobacteria remains unknown, our data highlight the possibility of coevolution of sulfate reduction and cyanobacterial anoxygenic photosynthesis in microbial mat systems where local sulfur cycling is fueled by a dense biofilm population.

## Figures and Tables

**Table 1:** Statistics for the *Leptolyngbya* sp. strain hensonii draft genome

Scaffolds	77
Longest scaffold (bp)	544 817
	
*General information*	
Total bp	5 940 030
N50 (bp)	137 782
	
*Characteristics*	
G+C	52.32
tRNA	61
Protein coding genes	5627

**Table 2:** Definition of the rate laws governing the redox reactions shown in Figure 1

*Expression*	*Description*
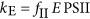	The rate of generation of an excited catalytic Chlorophyll *a* (Chl *a*) dimer in photosystem II (PSII*). It depends on irradiance (*E*), the availability of the ground state Chl *a* in PSII (PSII) and the absorbance cross-section factor *f*_II_ that describes the efficiency of conversion of the externally available photon flux (*E*) into a volumetric rate of excitation
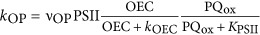	The rate of PQ reduction by PSII, that is, the rate of oxygenic photosynthetic electron transport. It depends on the availability of the excited catalytic Chl *a* dimer in PSII (PSII*), non-inhibited oxygen evolving complex (OEC) and oxidized plastoquinone (PQ_ox_). This process results in the formation of a highly reactive oxidized oxygen evolving complex (OEC_ox_), reduced plastoquinone (PQ_red_) and regeneration of ground state Chl *a* in PSII (PSII)
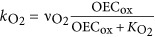	The rate of O_2_ release from the OEC, which depends on the availability of oxidized OEC (OEC_ox_)
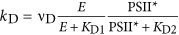	The rate of PSII degradation by photoinhibition. It depends on the availability of the excited catalytic Chl *a* dimer in PSII (PSII*) and the irradiance (*E*). To account for light-dependent efficiency of photoinhibition the rate saturates at high light intensities
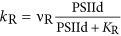	The rate of repair of the partially degraded, non-active PSII (PSIId)
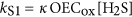	The rate of OEC_ox_ inhibition by H_2_S. This rate depends on the availability of the intermediate formed during OEC oxidation (OEC_ox_) and yields OEC:H_2_S, which refers to H_2_S being bound to OEC_ox_
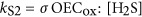	The rate of OEC_ox_ release from OEC_ox_:H_2_S, that is, the rate of deinhibition
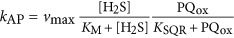	The rate of H_2_S oxidation coupled to PQ_ox_ reduction by SQR, that is, the rate of anoxygenic photosynthetic electron transport. This process results in the formation of zero-valent sulfur and PQ_red_
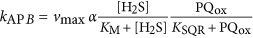	Assuming that a hypothetical additional sulfide-oxidizing enzyme (see ‘USO’ in *k*_AP2_) exists or that two SQRs exist that are expressed dependent on the light intensity (not shown), this rate is *k*_AP_ and depends exclusively on the H_2_S concentration ([H_2_S]) and the availability of PQ_ox_
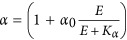	Assuming that the activity of SQR is directly regulated by the light intensity the maximum rate of H_2_S oxidation this rate is *k*_AP_B_. In this rate *ν*_max_, additionally depends on a factor *α* that increases with irradiance (*E*)
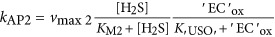	The rate of H_2_S oxidation coupled to the reduction of another electron transport chain component (‘EC’), such as oxidized cytochrome *c*, by a hypothetical sulfide oxidizing enzyme ‘USO’. This rate is only included in the model when assuming that the activity of SQR is *not* directly regulated by the light intensity (see description of k_AP_ and k_AP_B_)
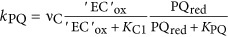	The rate of PQ_red_ oxidation coupled to the reduction of any electron transport chain component (‘EC’). This process results in the reformation of PQ_ox_, which is available again for the reduction by SQR or PSII
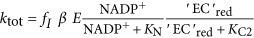 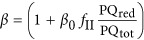	The rate of NADP^+^ reduction coupled to the oxidation of the unidentified electron transport component ‘EC’. This rate depends on the availability of reduced ‘EC’, NADP^+^, irradiance (*I*_PSI_ in μmol photons m^−2^ s^−1^), and the absorbance cross-section factor *f*_*I*_ that describes the efficiency of conversion of the externally available photon flux (*E*) into a volumetric rate of excitation in PSI. *f*_I_ is increased by excitation energy transfer from PSII to PSI, which is described by the transfer factor *β*. *β* depends on the redox state of the plastoquinone pool where PQ_red_ refers to the reduced part of the total PQ pool (PQ_tot_)
	
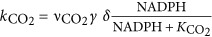 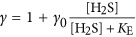 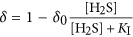	The rate of CO_2_ fixation coupled to NADPH oxidation, which depends on the maximum rate of CO_2_ reduction ( 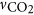 ) and the availability of NADPH. 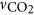 is enhanced when H_2_S available, which is described by the factor *γ*. At high H_2_S concentrations (*K*_E_<<*K*_I_) 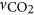 decreases again, which is described by the factor *δ*
