# Supplementary material for: Cyanobacterial photosynthesis under sulfidic conditions: insights from the isolate Leptolyngbya sp. strain hensonii
Source: ISME J. 2018 Jan 12;12(2):568–84. doi: 10.1038/ismej.2017.193 (PMC5776472; doi:10.1038/ismej.2017.193)
Supplement: Supplementary Material [file ismej2017193x1.pdf]

Cyanobacterial photosynthesis under sulfidic conditions - Insights from the isolate *Leptolyngbya* sp. strain hensonii

Trinity L. Hamilton, Judith M. Klatt, Dirk de Beer, and Jennifer L. Macalady

SUPPLEMENTAL MATERIAL

CONTENTS

1. SUPPLEMENTAL METHODS 2
   1.1. Enrichment and isolation 2
   1.2. Nucleic acid analyses 2
   1.3. Genomic sequencing 2
   1.4. Phylogenetic analysis 3
2. MODEL FOR THE KINETIC REGULATION OF OXYGENIC AND ANOXYGENIC PHOTOSYNTHESIS 4
   2.1. Oxygenic photosynthetic electron transport reactions 4
      2.1.1. Formulation of Rate laws for oxygenic photosynthesis 5
   2.2. Anoxygenic electron transport reactions 10
      2.2.1. Formulations of rate laws for anoxygenic photosynthetic electron transport 10
3. SUPPLEMENTAL FIGURES 14
4. SUPPLEMENTAL TABLES 20
5. SUPPLEMENTAL REFERENCES 26

1. SUPPLEMENTAL METHODS

1.1. Enrichment and isolation

Samples of red pinnacle mat were homogenized and a small aliquot (~ 500 µL) was added to BG11 media (Rippka et al., 1979) supplemented with 25 mM HEPES (B-HEPES) and adjusted to pH 7.2. Enrichment cultures were maintained in 60-mL of liquid media in 125-mL conical flasks at 100 RPM at 28 ºC under either a day-night cycle or continuously illuminated with 100 µmol photons m^{-2} s^{-1} under cool white fluorescent lamps. An axenic culture was achieved using a dilution series in liquid media where the highest dilution which showed growth was taken as inoculum for the next dilution. Light microscopy was performed periodically to visually examine enrichments for purity. The dilution to extinction strategy was continued until light microscopy indicated the presence of a single morphotype and sequencing of the 16S rRNA gene returned a single phylotype. Growth of the isolate was monitored with chlorophyll *a* concentration determined spectrophotometrically using the absorption at 665 nm of a methanol extract and an extinction coefficient of 0.075 ml μg^{-1} (made from a filtered 2-ml culture subsample) (De Marsac and Houmard, 1988) or protein concentration using the Bradford assay (Bradford, 1976) with bovine serum albumin (Sigma-Aldrich, St. Louis, MO) as the standard.

1.2. Nucleic acid analyses

Samples of biofilm (~ 1.5 mL) were harvested by centrifugation, the excess media removed by decanting, and cell pellets frozen immediately (-20 °C) or subjected to nucleic acid extraction. Genomic DNA was extracted as previously described (Boyd et al., 2007). Quality of extracted DNA was assessed on an agarose gel (1%) using the HiLo DNA Marker (Bionexus, Oakland, CA) and visualized by ethidium bromide staining and using a NanoDrop ND-1000 spectrophotometer (NanoDrop Technologies, Wilmington, Delaware). To check for purity/contaminants, 16S small subunit RNA genes were amplified with bacterial domain primers 27F and 1492R were used (Lane, 1991) as described previously (Hamilton et al., submitted). Reactions were performed in triplicate, purified using a QIAquick PCR Purification Kit (Qiagen, Valencia, CA) and sequenced at the Genomics Core Facility of the Huck Institutes of the Life Sciences at Penn State University. Sequences were assembled and manually checked using using Bio-Edit (v7.2.5), and checked for chimeras using CHIMERA_CHECK (Cole et al., 2003). Putative chimeras were excluded from subsequent analyses. A single 16S rRNA gene sequence was recovered indicating the culture was pure.

1.3. Genomic sequencing

Double-stranded gDNA was quantified by Qubit (Life Technologies, Carlsbad, CA) and 100 ngs of DNA was fragmented for 7 minutes at 37 ºC using the Ion Shear Reagent according to the Ion Xpress™ Plus gDNA and Amplicon Library Preparation Users Guide (Life Technologies, Carlsbad, CA). Fragments

were purified using AMPure XP Reagent (Beckman Coulter, Brea, CA) and checked for size on a Bioanalyzer DNA High Sensitivity chip (Agilent Technologies, Santa Clara, CA). Adaptors were ligated to the fragments and DNA was nicked, repaired and then purified. A 315 bp library was size-selected on a Pippin (Sage Science, Beverly MA) using the "tight setting". Following purification, the library was checked again on the Bioanalyzer and then amplified on beads in an emulsion PCR using the Ion Xpress Template Kit and 200 base sequencing was performed using a 316 chip on the Ion PGM machine as per the Users Guide (Life Technologies, Carlsbad, CA) at the Genomics Core Facility of the Huck Institutes of the Life Sciences at Penn State University.

1.4. Phylogenetic analysis

For gene-specific phylogenetic analyses, full-length sequences were identified in the genome using functional annotation and BLASTX, translated, and verified by BLASTP. Reference datasets were populated by detecting homologs in IMG genomic databases by BLASTP (Altschul et al., 1997). Protein sequences were aligned with MUSCLE (Edgar, 2004) and redundancy in the alignments was reduced through the Decrease Redundancy Program (http://web.expasy.org/decrease_redundancy/). Maximum likelihood trees were constructed using PhyML (Guindon et al., 2010) with the LG+gamma model, four gamma rate categories, ten random starting trees, NNI branch swapping, and substitution parameters estimated from the data. The resulting trees were viewed and edited using iTOL (http://itol.embl.de/) (Letunic and Bork, 2016).

2. MODEL FOR THE KINETIC REGULATION OF OXYGENIC AND ANOXYGENIC PHOTOSYNTHESIS

Leptolyngbya sp. strain hensonii shows a complex response to H_2S: (I) rates of anoxygenic photosynthesis (AP) are regulated by both H_2S and irradiance (Fig. 5); (II) O_2 production is entirely inhibited by minute H_2S concentrations (Fig. 4); (III) the recovery rate of oxygenic photosynthesis (OP) after inhibition is dependent on irradiance (Fig. 6); and (IV) AP rates can be double those of OP (Fig. 3; Fig. 5). To constrain the range of possible biochemical mechanisms shaping this response, we constructed a simplified model of the electron transport reactions involved in oxygenic and anoxygenic photosynthesis. The model described here represents a fusion and further developed version of models described in Klatt *et al.*, 2015a, and 2015b.

Using this model we show that the inhibition and recovery of OP can be explained based on the interaction of H_2S with an intermediate of the oxygen evolving complex (OEC), the generation of which is light dependent. Also, we show that the regulation of AP is substantially more complex than in previously studied cyanobacterial strains and cannot be explained by just considering the properties of SQR, i.e., its affinity for oxidized PQ and H_2S. Instead, we suggest that H_2S affects reactions downstream of PSI and we present two independent hypotheses concerning the light-dependency of the kinetics of H_2S oxidation: (a) light-dependent activity of SQR and (b) multiple H_2S oxidizing enzymes.

2.1. Oxygenic photosynthetic electron transport reactions

Cyanobacteria perform OP via electron transport reactions that are often represented as the Z-scheme (Fig. S3) and that have been intensively reviewed (reviewed e.g. in (Vermaas 2001; Govindjee et al., 2010; Shevela et al., 2013; Roach and Krieger-Liszkay, 2014; Johnson 2016)). In brief, photosynthesis is initiated by the absorption of photons by pigments associated with PSII and PSI. The light energy is absorbed by accessory (also: antennae) pigments, such as phycocyanin, phycoerithrin, and other chlorophylls, is then transferred in the form of excitation energy to the reaction center (RC) chlorophyll *a* (Chl *a*). Via various intermediate redox reactions the excited state reaction center Chls drive the oxidation of the external electron donor H_2O and the reduction of redox carriers $NADP^+$. During electron transport a proton motive force (pmf) is generated, which drives the formation of ATP. Thus, during these "light reactions" of this linearly organized electron transport chain the excitation energy is converted into chemical energy in the form of reduced electron carriers and ATP. The NADPH and ATP generated during these electron transport chain reactions are driving CO_2 fixation in the "dark" reactions of the Calvin cycle. The individual reactions involved in oxygenic photosynthetic electron transport are described in greater detail below.

As cyanobacteria are often exposed to fluctuating environmental conditions, the photosynthetic electron transport and its regulation are complex (reviewed e.g. in (Peschek, 1999; Vermaas, 2001; Mullineaux 2014a; Nagarajan *et al.*, 2015). There are multiple possibilities for alternative electron flow,

i.e., “branching” of the main electron transport highway. The “valves” of the linearly organized photosynthetic electron transport chain become important regulators when otherwise excessive oxidation or reduction of the transport chain components would occur.

Most prominently, the PQ pool, the cytochrome b_6f complex, and the soluble electron carriers (plastocyanin or cytochrome c_{553}) are also shared with the respiratory pathway (Fig. S3). PQ in the thylakoid membranes can receive electrons from succinate dehydrogenase (SDH). Reduced PQ, plastoquinol, can directly donate electrons to terminal oxidases, such as the plastoquinol oxidase (PTOX), the alternative respiratory terminal oxidase (ARTO) and cytochrome *bd*-type quinol oxidase (Cyd) (Lea-Smith et al., 2016). These oxidases couple the oxidation of PQ to the reduction of O_2. In addition, the soluble cytochromes can donate electrons to a terminal oxidase, aa_3-type cytochrome-c oxidase complex, instead of PSI. It is important to note for the discussion below that genes encoding both a cytochrome *c* oxidase and a *bd*-type quinol oxidase were identified in the strain hensonii genome. PQ reduction by SDH and PQ (or cytochrome) oxidation via terminal oxidases can likely occur simultaneously with photosynthetic electron transport, with O_2 reduction representing an electron “dump”. These reactions are likely important for cyanobacteria to maintain redox balance and prevent cell damage by photoinhibition but can also be dangerous (Murata et al., 2012). Cyclic electron transport can also occur via electron transfer from Ferredoxin to PQ. Furthermore, PSI and PSII have several other alternative electron acceptors, such as Flavodiiron proteins (Flv1-4) and a bidirectional [NiFe] hydrogenase (Hox) (Zhang *et al.*, 2012; Gutekunst *et al.*, 2014; Mullineaux, 2014b).

While possibly representing important regulatory functions to maintain redox balance, we assume here for simplicity that the rates of cyclic electron transport and other alternative electron flow reactions are insignificant compared to the photosynthetic electron mainstream reactions. Our methods do not allow for a quantification of the rates of these reactions and, as demonstrated below, their consideration is not needed to explain our data. We therefore limit our simplified model to the main components of the electron transport chain. Furthermore, we only concentrate on the reaction rates that can become the kinetic bottleneck for photosynthetic electron transport.

2.1.1. Formulation of Rate laws for oxygenic photosynthesis

In our model we assume that during non-saturating light conditions, light energy harvested in PSI or PSII limits OP, as manifest in the linear increase of OP with light in PI-curves (Fig. 7) (Klatt *et al.*, 2015b). Thus, the light energy harvested by accessory pigments that has migrated to the Chl *a* in the reaction center of PSII determines the rate (k_E) of generation of an excited catalytic Chl *a* dimer in PSII (PSII*). This can be formulated as

$$k_E = f_{II}\ E\ \text{PSII} \tag{S1}$$

where PSII is ground state Chl *a* in PSII and f_{II} is an absorbance cross-section factor that describes the efficiency of conversion of the externally available photon flux (E) into a volumetric rate of excitation. Upon the generation of an excited Chl *a* in PSII, charge separation between this Chl and primary electron

acceptor pheophytin (Pheo) occurs. The term charge separation describes the formation of a radical pair, P680•+ and Pheo•- in PSII. This is the initiation of the electron transport reactions. Pheo rapidly reduces a bound plastoquinone (Q_A), which donates electrons to a next redox carrier, another plastoquinone (Q_B), which joins the mobile plastoquinone pool (PQ). While the initial reactions are extremely fast and occur directly in PSII RCs, PQ reduction and mobilization, and the following re-oxidation by cyt b_6f are comparatively slow, which is related to the diffusion time of the PQ molecules in the thylakoid membranes (Whitmarsh and Govindjee, 1999). As a result, the redox state of the PQ pool is highly sensitive to imbalances between energy harvested in PSI and II, and to the rate saturation downstream of PSI. In other words, PQ is an important indicator of the overall redox state of the photosynthetic electron transport chain. Importantly, the availability of oxidized PQ can become the rate limiting factor for OP.

In our model the rate of electron transport initialization is, however, not only dependent on the availability of oxidized PQ but also to the availability of a functional oxygen evolving complex (OEC) that is not inhibited by H_2S and can mediate the oxidation of H_2O to O_2. As mentioned above, charge separation leads to the formation of an oxidized RC Chl *a* (P680+). Electrons are rapidly transferred from a tyrosine residue of the OEC, which is in turn reduced by the Mn_4O_xCa cluster of the OEC. Overall, the rate of PQ reduction by PSII (k_{OP}) can thus be described as following Michaelis-Menten kinetics according to

$$k_{OP} = \nu_{OP}\ \mathrm{PSII}^* \frac{\mathrm{OEC}}{\mathrm{OEC} + K_{OEC}} \frac{\mathrm{PQ_{ox}}}{\mathrm{PQ_{ox}} + K_{PSII}} \tag{S2}$$

where PSII* is the excited catalytic Chl *a* dimer in PSII, OEC is non-inhibited oxygen evolving complex and PQ_{ox} is the oxidized part of the plastoquinone pool. v_{OP} is the maximum rate and K_{OEC} and K_{PSII} are the reaction constants describing the apparent affinity of PSII* towards OEC and oxidized PQ.

To oxidize two water molecules and generate one O_2 molecule, four oxidizing equivalents in the Mn_4O_xCa cluster have to be generated ultimately driven by 4 photons harvested by PSII. The exact reactions occurring in the OEC are still to be fully described (Messinger and Renger, 2008; Jablonsky and Lazar, 2008). Clearly, the OEC undergoes several transitional stages (initially described as S states; (Kok *et al.*, 1970)) before release of an O_2 molecule. In our model, we use "OEC_{ox}" to summarize multiple intermediate stages of the OEC. The process described by k_{OP} in Eq. S2 results in the formation of the oxidizing equivalents OEC_{ox}. The rate of O_2 release from the OEC will depend on the availability of OEC_{ox} which can be described as

$$k_{O2} = \nu_{O2} \frac{\mathrm{OEC_{ox}}}{\mathrm{OEC_{ox}} + K_{O2}}\ . \tag{S3}$$

Klatt *et al.* (2015b) hypothesized that the inhibitory effects of H_2S is based on interaction with an intermediate of the OEC, OEC_{ox}, according to

$$k_{S1} = \kappa\ \mathrm{OEC_{ox}}[\mathrm{H_2S}]\ . \tag{S4}$$

De-inhibition kinetics, i.e. the rate of release of H_2S from the OEC, can be described as

$$k_{S2} = \sigma \ \mathrm{OEC_{ox}:[H_2S]} \qquad \text{(S5)}$$

where OEC:[H_2S] describes the inhibited state of the OEC.

The reduction of PQ coupled to oxidation of OEC described by Eq. S2 regenerates ground state Chl *a* in PSII. If insufficient PQ_{ox} or OEC are available, PSII* consequently accumulates causing enhanced photoinhibition, i.e., degradation of the D1 subunit of PSII. In our model, we describe photoinhibition as PSII degradation upon an additional photon harvesting event in the presence of PSII* (Nishiyama et al., 2006). The rate of PSII degradation will therefore depend on the availability of the excited catalytic Chl *a* dimer in PSII (PSII*) and irradiance (E) according to

$$k_D = \nu_D \frac{E}{E + K_{D1}} \frac{\mathrm{PSII}^*}{\mathrm{PSII}^* + K_{D2}} . \qquad \text{(S6)}$$

K_{D1} was chosen such that the rate begins to saturate at high light intensities because we also assumed that the efficiency of photoinhibition is light-dependent (Keren et al., 1997). Degraded subunits of PSII are repaired at a rate described by

$$k_R = \nu_R \frac{\mathrm{PSIId}}{\mathrm{PSIId} + K_R} \qquad \text{(S7)}$$

where PSIId is non-active PSII. This reaction leads to the replenishment of active ground-state PSII.

The successful initialization of electron transport yields reduced PQ according to Eq. S2. Oxidation of PQ occurs via the cyt b_6f complex and plastocyanin (or cytochrome c_{553}), and is driven by light energy harvested in PSI. From PSI electrons are transferred via a series of fast intermediate redox reactions to ferredoxin, which, via ferredoxin-NADP$^+$ oxidoreductase (FNR), can reduce NADP$^+$. The rate of PQ oxidation can thus be described as

$$k_{tot} = f_I \ \beta \ E \frac{\mathrm{NADP}^+}{\mathrm{NADP}^+ + K_N} \frac{\mathrm{PQ_{red}}}{\mathrm{PQ_{red}} + K_{C3}} \text{, with} \qquad \text{(S8)}$$

$$\beta = (1 + \beta_0 \ f_{II} \frac{\mathrm{PQ_{red}}}{\mathrm{PQ_{tot}}}) \qquad \text{(S9)}$$

where f_I is an absorbance cross-section factor of PSI. The factor β introduces excitation energy transfer from PSII to PSI dependent on redox state of the PQ pool. Namely, with increasing reduced PQ (PQ_{red}) in the total PQ pool (PQ_{tot}) an increasing fraction β of light energy harvested in PSII is transferred to PSI, which represents a mechanism to prevent an electron "traffic jam".

The process described by Eq. S8 yields oxidized PQ that is available again for reduction by PSII and NADPH. The redox carrier NADPH is used in the Calvin cycle for CO_2 reduction. The rate of CO_2 fixation coupled to NADPH oxidation, which depends on the maximum rate of CO_2 reduction, and the availability of NADPH, which can be formulated as

$$k_{CO2} = \nu_{CO2} \frac{\text{NADPH}}{\text{NADPH} + K_{CO2}} . \qquad \text{(S10)}$$

The model for oxygenic photosynthetic electron transport is solved by finding steady state solutions to six differential equations:

$$\frac{d\text{PSII}^*}{d\text{t}} = k_E - k_{OP} - k_D \qquad \text{(S11)}$$

describes the rate of change in the fraction of RC Chl *a* that is in excited state. PSII* is generated at the rate k_E (Eq. S1) and decreases due to degradation of PSII* and by OEC reduction at rates k_D and k_{OP}, respectively. The ground state fraction of PSII is generated at a rate described as

$$\frac{d\text{PSII}}{d\text{t}} = k_{OP} + k_R - k_E \qquad \text{(S12)}$$

as a result of OEC oxidation coupled to PQ reduction (k_{OP}) and repair of degraded PSII (k_R). The rate of PSII decrease by excitation is k_E. Importantly, the sum of all fractions of photosystem II are constant in our model, which can be written as

$PSII_{tot}$ = PSII + PSII* + PSIId. (S13)

The fraction of unstable intermediately oxidized OEC (OEC_{ox}) in the total OEC pool,

OEC_{tot} = OEC + OEC_{ox} + OEC_{ox}:H_2S, (S14)

is increasing during re-establishment of ground state PSII and PQ reduction (k_{OP}) and "de-inhibition" by H_2S (k_{S2}), and decreasing due to inhibition (k_{S1}) and H_2O oxidation (k_{O2}) The rate of change of the oxidized fraction of OEC (OEC_{ox}) is thus described as

$$\frac{d\text{OEC}_{\text{ox}}}{d\text{t}} = k_{OP} + k_{S2} - k_{O2} - k_{S1} , \qquad \text{(S15)}$$

while the rate of change of the fraction of OEC in the total OEC pool follows

$$\frac{d\text{OEC}}{d\text{t}} = k_{O2} - k_{OP} , \qquad \text{(S16)}$$

which describes that OEC is a result of H_2O oxidation (k_{OP}), and regeneration of PSII and reduction of PQ (k_{OP}). Similarly, the differential equation

$$\frac{d\text{PQ}_{\text{ox}}}{d\text{t}} = k_{tot} - k_{OP} \qquad \text{(S17)}$$

describes the rate of change of the oxidized fraction of the PQ pool (PQ_{ox}) as the result of PQ reduction by PSII* (k_{OP}) and PQ oxidation driven by light in PSI, coupled to $NADP^+$ reduction (k_{tot}). The sum of the different fractions of the PQ pool are again set constant, i.e.,

$PQ_{tot} = PQ_{red} + PQ_{ox}$, (S18)

which also applies to the total $NADP^+$/NADPH pool ($NADP_{tot}$), i.e.,

$NADP_{tot} = NADP^+ + NADPH.$ (S19)

In our model, NADPH is generated during PQ oxidation (k_{tot}) and consumed by CO_2 fixation (k_{CO2}), which is described as

$$\frac{d\mathrm{NADPH}}{d\mathrm{t}} = k_{tot} - k_{CO2} \quad \text{(S20)}$$

By setting dPSII*/dt = 0, dPSII/dt = 0, OEC_{ox}/dt = 0, dOEC/dt = 0, dPQ_{ox} = 0 and dNADPH/dt = 0, the steady-state redox state of the PSII, OEC, PQ and NADP pool and the corresponding rate of oxygenic photosynthetic electron transport can be calculated using Eqs. S1 – S11 for any given values of [H_2S] and E.

Our data was fitted using a numerical implementation of the model into R with the deSolve package (Soetaert et al., 2010). When testing reasonable values for the maximum rates v_x, affinity constants K_x and for the factors f_I and f_{II} in Eqs. S1 – S10 we considered that at saturating light intensities, rates of OP stop increasing linearly with light (Fig. 7) and rates of CO_2 fixation are expected to become the bottleneck of OP rates. Therefore, K_{CO2} and v_{CO2} in Eq. S10 had to be set substantially higher than all other maximum rates and affinity constants to achieve that k_{CO2} limits the overall electron transport at high light only. We also considered that in most cyanobacteria PSI RCs are more abundant in the thylakoid membrane than PSIIs to prevent that k_{tot} becomes the bottleneck for electron transport because an electron transport "traffic jam" can cause photodamage to PSII, as described above. We therefore set $f_I > f_{II}$, which revealed very good agreement with the experimental data (see dashed line in Fig. 7).

In the formulation of OEC_{ox} inhibition by H_2S (Eq. S4), the rate of inhibition will be dependent on light because the abundance of OEC_{ox} is light-dependent (see Eq. S1 and S2). Klatt *et al.* (2015b) have indeed observed pronounced light-dependent inhibition kinetics in their studied *Planktothrix* strain. We could not observe similar kinetics in *Leptolyngbya* sp. strain hensonii — H_2S instantaneously inhibited OP at very low concentrations. Therefore, κ was chosen to be substantially higher than in the model for the cyanobacteria previously described in Klatt et al. (2015b).

After exposure to H_2S, oxygenic photosynthetic rates only started to recover after a fixed time frame of ~30 min. This could be explained by considering in our model that $\sigma <<< \kappa$ in Eqs. S4 and S5, which implies that any available OEC_{ox} is rapidly inhibited by H_2S, while the back reaction is so slow that the inhibited state of the OEC dominates for an extended time frame even after removal of externally available H_2S. Furthermore, the recovery rate of oxygenic photosynthesis from H_2S inhibition was light-dependent in strain hensonii (Fig. 6). Inhibition of photosynthesis by H_2S on the donor side of PSII, is expected to cause photodamage of PSII (Jegerschoeld et al., 1990) because the excitation energy cannot be converted into chemical energy in the form of electron transport. We adjusted constants in our model such that an active OEC is only slowly re-generated even after depletion of H_2S. Thus, excited/oxidized RC Chl *a* (PSII*) is more abundant than without "H_2S-history" and rates are highly dependent on light (E) excuse light determines degradation rates (Eq. S6). The output of our model was in remarkable

agreement with the experimental data (see black lines in Fig. 6). We therefore conclude that the light-dependent recovery rates can be explained based on the intertwined kinetics of OEC inhibition by H_2S and photoinhibition. i.e., light-dependent degradation of D1 in PSII.

2.2. Anoxygenic electron transport reactions

The model considers that in photosynthetically versatile cyanobacteria, electrons derived from H_2S oxidation enter the electron transport chain at the level of the PQ pool via SQR (Fig. S3). Thus, the electron transport pathways of AP and OP intersect in the PQ pool. Cyanobacteria therefore use all redox active compounds downstream of PQ, towards $NADP^+$ and ultimately to CO_2, for the transport of both H_2S- and H_2O-derived electrons. It is thus a crucial assumption in our model that the kinetics of photosynthetic electron transport beyond PQ are independent of photosynthetic mode unless direct modifications by H_2S occur.

2.2.1. Formulations of rate laws for anoxygenic photosynthetic electron transport

The rate of H_2S oxidation is expected to be dependent on the affinity of SQR to H_2S and to PQ, which can be formulated as

$$k_{AP} = v_{\max} \frac{[\mathrm{H_2S}]}{K_M + [\mathrm{H_2S}]} \frac{\mathrm{PQ_{ox}}}{K_{SQR} + \mathrm{PQ_{ox}}} \qquad \text{(S21)}$$

according to Klatt et al. (2015a). The differential equation S17 thus changes to

$$\frac{d\mathrm{PQ_{ox}}}{d\mathrm{t}} = k_{tot} - k_{OP} - k_{AP} \quad . \qquad \text{(S22)}$$

The rate of AP depends on the availability of H_2S and redox status of the PQ pool.

In our model all rates are in µM electrons s^{-1}. The rate of H_2S depletion by AP can be calculated as

$$\frac{d\mathrm{H_2}S}{d\mathrm{t}} = 0.5 k_{AP} \qquad \text{(S23)}$$

by considering that H_2S is oxidized to zero-valent sulfur according to $H_2S \rightarrow S^0 + 2\ e^-$. The rate of total sulfide depletion can be derived from the change of H_2S concentration at every time point by considering the pH according to

$$[\mathrm{S_{tot}}] = [\mathrm{H_2S}](1 + \frac{\mathrm{K_1}}{[\mathrm{H_3O}]}) \quad . \qquad \text{(S24)}$$

H_2S entirely inhibits the OEC and thus the reduction of PQ by PSII. Consequently, PQ redox does not depend on competitive reductive reactions but on H_2S availability and the bottleneck reactions downstream of PQ, which determine the re-oxidation rate of PQ. Under non-saturating light conditions the bottleneck is light harvested in PSI. At high light intensities other reactions downstream of PSI namely CO_2 fixation (k_{CO2}, Eq. S10) might become the bottleneck of photosynthetic reaction thus

ultimately governing also the rate of H_2S oxidation. We observed that electron transport rates of AP were double those of OP at high light conditions (Fig. 5; Fig. 7). High H_2S concentration, however, caused a decrease of rates (Fig. 5). We therefore reformulated Eq. S10 to

$$k_{CO2} = \nu_{CO2}\, \gamma\, \delta \frac{\text{NADPH}}{\text{NADPH} + K_{CO2}}, \tag{S23}$$

where

$$\gamma = 1 + \gamma_0 \frac{[\text{H}_2\text{S}]}{[\text{H}_2\text{S}] + K_E}, \tag{S24}$$

which describes the enhancement of maximum rates of CO_2 fixation with H_2S, and where

$$\delta = 1 - \delta_0 \frac{[\text{H}_2\text{S}]}{[\text{H}_2\text{S}] + K_I}, \tag{S25}$$

which describes the inhibition of CO_2 fixation by H_2S. By setting $K_E < K_I$, the enhancement of rates is pronounced at low [H_2S] and is outweighed by the inhibitory effect at higher [H_2S]. Also, CO_2 fixation only becomes the bottleneck for electron transport at high light intensities E. Thus, the modulation of electron transport rates by H_2S also only become apparent at high light intensities. At lower light intensities, AP strictly depends on [H_2S], PQ redox and light harvested in PSI.

The numerical implementation of this model gave excellent results for the enhancement and inhibition of AP at higher E. However, we could not explain the light dependent slope of rates of AP in Fig. 5. This is because our model predicts that rates of AP are increasing with [H_2S] until a light-dependent maximum is reached, i.e., until PSI turns into the bottleneck for electron transport. Below this maximum, light does not affect rates of AP (Klatt et al., 2015a). We could only achieve an agreement of our model with the experimental data by using either of two assumptions: (i) Light modulates the activity of SQR by increasing v_{max} in Eq. S21 and (ii) there are two sulfide oxidizing enzymes that donate electrons into the electron transport chain at different levels. In the following, we introduce two different versions of the model based on these two assumptions.

2.2.1.1. Light-dependent v_{max}

The light dependency of rates of AP at low H_2S concentrations could be explained by light-dependent activity of SQR. This could be, for instance, due to light-dependent expression of SQR or even due to multiple SQRs, the relative abundance of which is controlled by light. In any case, this can be modeled by introducing light-dependent changes of v_{max} in Eq. S21 as

$$k_{AP\_B} = v_{\max}\alpha \frac{[\text{H}_2\text{S}]}{K_M + [\text{H}_2\text{S}]} \frac{\text{PQ}_{\text{ox}}}{K_{SQR} + \text{PQ}_{\text{ox}}}, \tag{S26}$$

where α increases with E according to

$$\alpha = (1 + \alpha_0 \frac{E}{E + K_\alpha}). \tag{S27}$$

The changes of H_2S oxidation rates in response to light were, however, instantaneous, which means that *hensonii* would possess a mechanism to continuously adjust the activity of SQR according to momentary irradiance. We considered this unlikely and thus considered an alternative explanation: the presence of a second, unidentified, sulfide oxidizing enzyme ("USO"). This assumption could explain our data as described below.

2.2.1.2. An additional sulfide oxidizing enzyme ("USO")

Assuming that a second sulfide oxidizing enzyme ("USO"), such as flavocytochrome c oxidase, exists, we implemented H_2S oxidation by this enzyme into our model as

$$k_{AP2} = v_{\max 2} \frac{[\mathrm{H_2S}]}{K_{M2} + [\mathrm{H_2S}]} \frac{\text{"EC"}_{\mathrm{ox}}}{K_{\text{"USO"}} + \text{"EC"}_{\mathrm{ox}}},$$

where "EC_{ox}" is any component of the electron transport chain between PQ and PSII. EC_{ox} thus receives electrons from both PQ and "USO". The rate of PQ oxidation (Eq. S8) thus changes to

$$k_{PQ} = \nu_C \frac{\text{"EC"}_{\mathrm{ox}}}{\text{"EC"}_{\mathrm{ox}} + K_{C1}} \frac{\mathrm{PQ_{red}}}{\mathrm{PQ_{red}} + K_{PQ}}$$

and is not directly coupled to reduction of $NADP^+$ anymore. $NADP^+$ reduction via PSI in our model is instead coupled to EC oxidation according to

$$k_{tot} = f_I \,\beta\, E \frac{\mathrm{NADP^+}}{\mathrm{NADP^+} + K_N} \frac{\text{"EC"}_{\mathrm{red}}}{\text{"EC"}_{\mathrm{red}} + K_{C2}},$$

where β is defined by Eq. S9.

Consequently, also the differential equation describing the rate of change of the oxidized fraction of the PQ pool (Eq. S22) had to be altered to

$$\frac{d\mathrm{PQ_{ox}}}{d\mathrm{t}} = k_{PQ} - k_{OP} - k_{AP}.$$

We additionally introduced the rate of change of the oxidized fraction of the EC pool as

$$\frac{d\text{"EC"}_{\mathrm{ox}}}{d\mathrm{t}} = k_{tot} - k_{OP} - k_{AP2}$$

and when finding a solution the sum of the total EC pool was constant as

$\text{"EC"}_{tot} = \text{"EC"}_{ox} + \text{"EC"}_{red}$.

The relative contribution of the two sulfide oxidizing enzymes to the overall sulfide oxidation activity will depend on their affinity to H_2S and the redox state of the respective electron acceptor in the transport chain. Assuming that "USO" donates electrons downstream of PQ it will cause its electron acceptor to become more reduced. This electron acceptor EC is also the electron acceptor for PQ. The activity of "USO" thus affects the oxidation state of PQ and thus SQR activity. At low light conditions competition for the electron transport chain takes effect. This competition scenario can occur when setting

K_{PQ} and K_{C1} > K_N and K_{C2}, which will lead to accumulation of reduced PQ at increasing H_2S concentrations. Fig. S4A shows that consequently rates of H_2S oxidation by SQR abruptly stop increasing at very low H_2S. This effect is most pronounced when setting the affinity constant of "USO" (K_{M2}) lower than that of SQR (K_M). At higher light, rates of overall electron transport through PSI are sufficiently high to keep both the reduced fraction of PQ and "EC" low enough to circumvent that PQ oxidation is limiting SQR activity at a low H_2S concentration (Fig. S4B). Then the rates of sulfide oxidation are strictly determined by the affinities to H_2S and not to the electron acceptors PQ and EC.

Our data set does not enable evaluation of both hypotheses. Regardless, the main conclusion of our modeling effort is that anoxygenic photosynthesis in *Leptolyngbya* sp. strain hensonii is controlled by substantially more complex mechanisms than expected based on previous studies, e.g. in *Planktothrix* FS39 described by Klatt et al. (2015a). Most prominently, rates of AP cannot be explained by assuming a steady pool of one single sulfide oxidizing enzyme.

3. SUPPLEMENTAL FIGURES

Figure S1. A). Schematic diagram of the experimental setup used in this study to measure rates of oxygenic and anoxygenic photosynthesis using microsensors. Cyanobacterial biofilms grown on glass fiber filters (a) were fixed on polyester fibrous web (b) that separated a top and bottom reservoir of media. After measurements of oxygenic photosynthesis in the uppermost layer of the biofilm, neutralized Na_2S solution was injected into both bottom and top reservoir and rates of oxygenic and anoxygenic photosynthesis, and the local H_2S concentration within the biofilm were monitored. A gentle flow of N_2 gas facilitated mixing. B). View looking down on the experimental setup with the oxygen, pH, and sulfide (dark) microsensors and the halogen lamps.

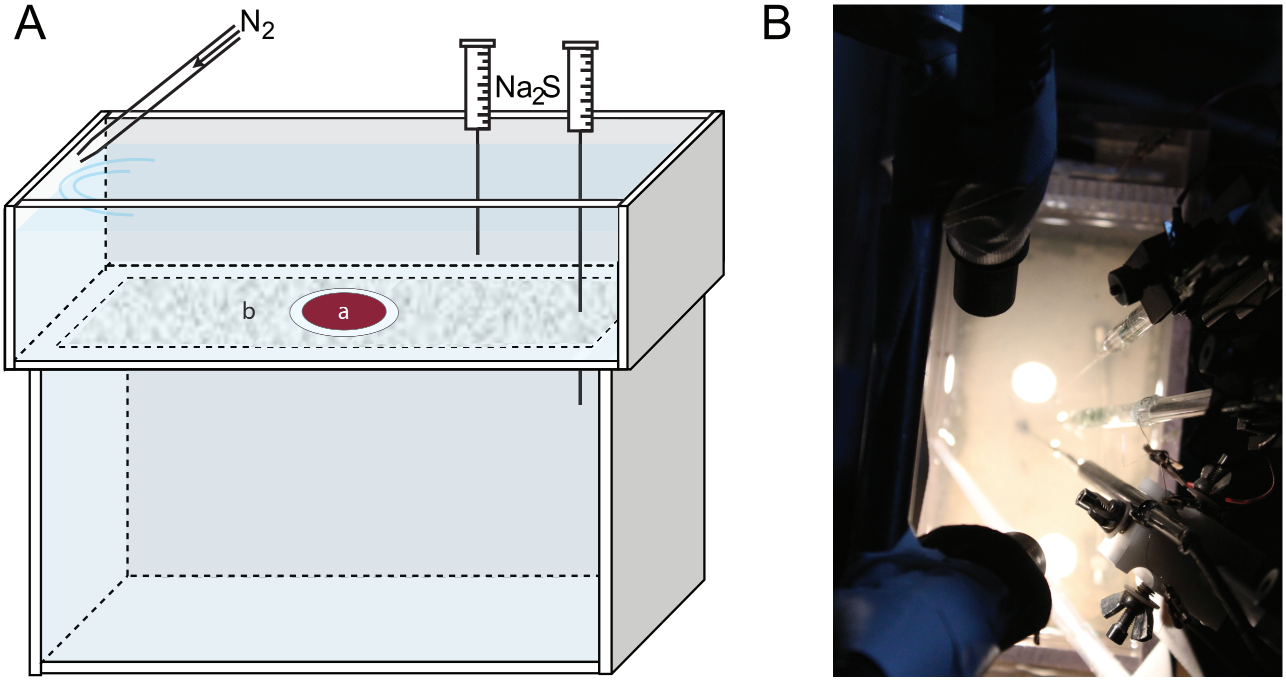

Figure S2. Light microscopy micrograph of strain hensonii.

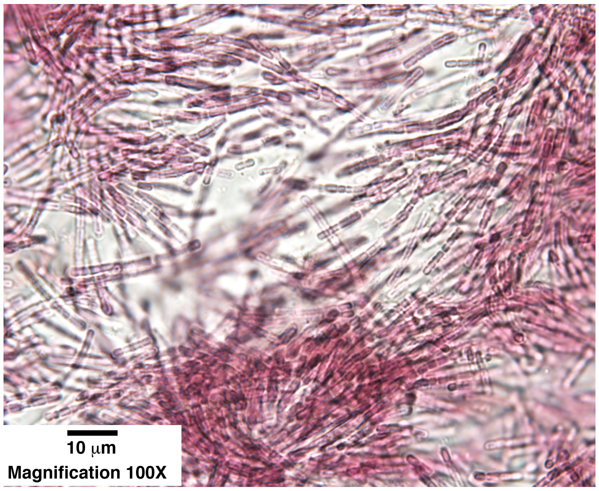

Figure S3. The electron transport chain of photosynthesis (the Z-scheme) showing the electron flow and the potential of redox pairs involved (A). The simplified Z-scheme used in model described here is shown in (B). Modified from Allen (2003) and Mullineaux (2014).

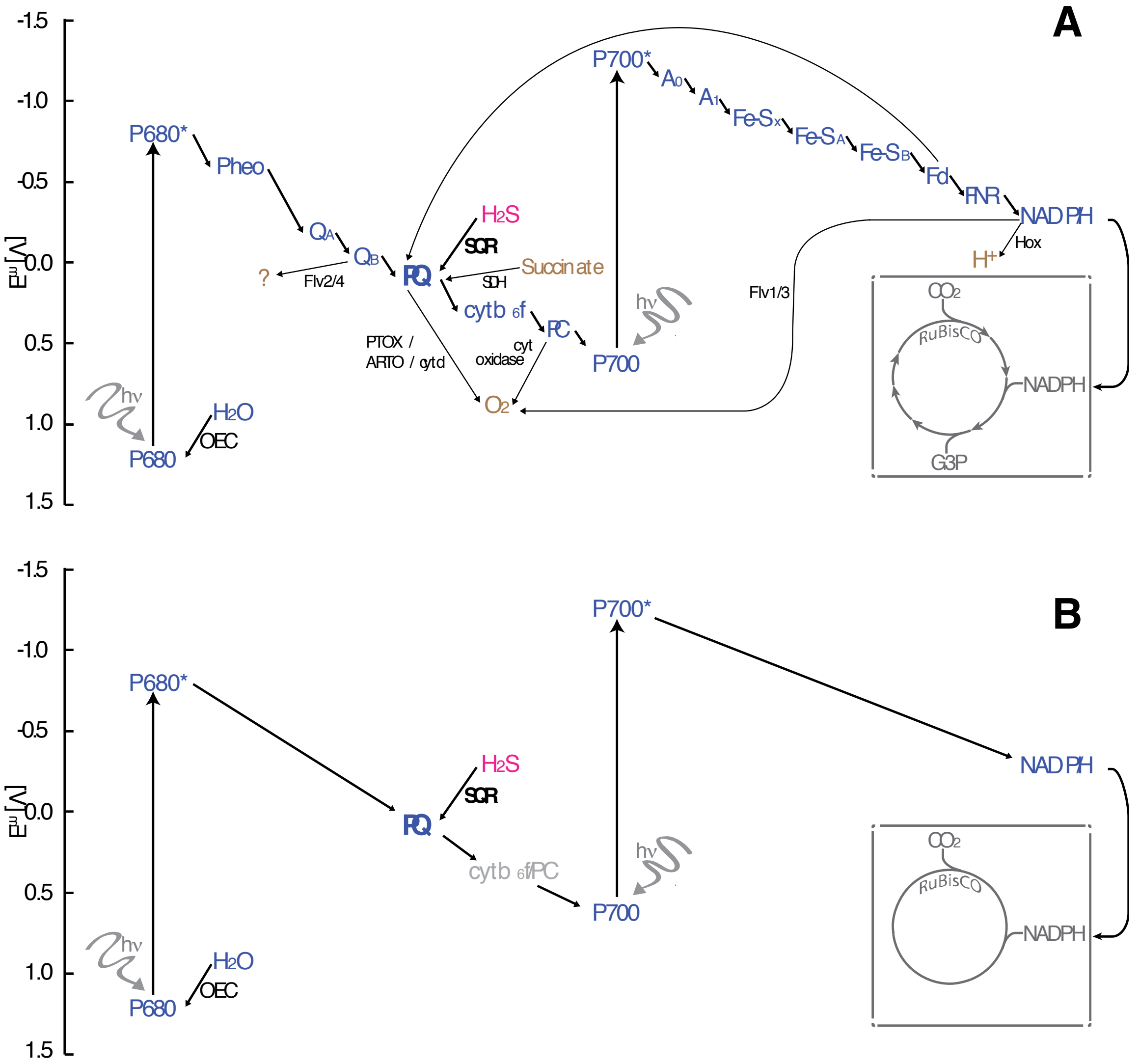

Figure S4. Modeled rates of electron transport resulting from H_2S oxidation by SQR (k_{AP}) and "USO" (k_{AP2}) expressed as % gross anoxygenic photosynthesis (GAP) of maximum oxygenic photosynthesis (GOP_{max}) dependent on H_2S concentration at 36 and 137 µmol photons m^{-2} s^{-1} (A and B, respectively). The steady state fraction of oxidized PQ and EC are also shown in % of the total pools.

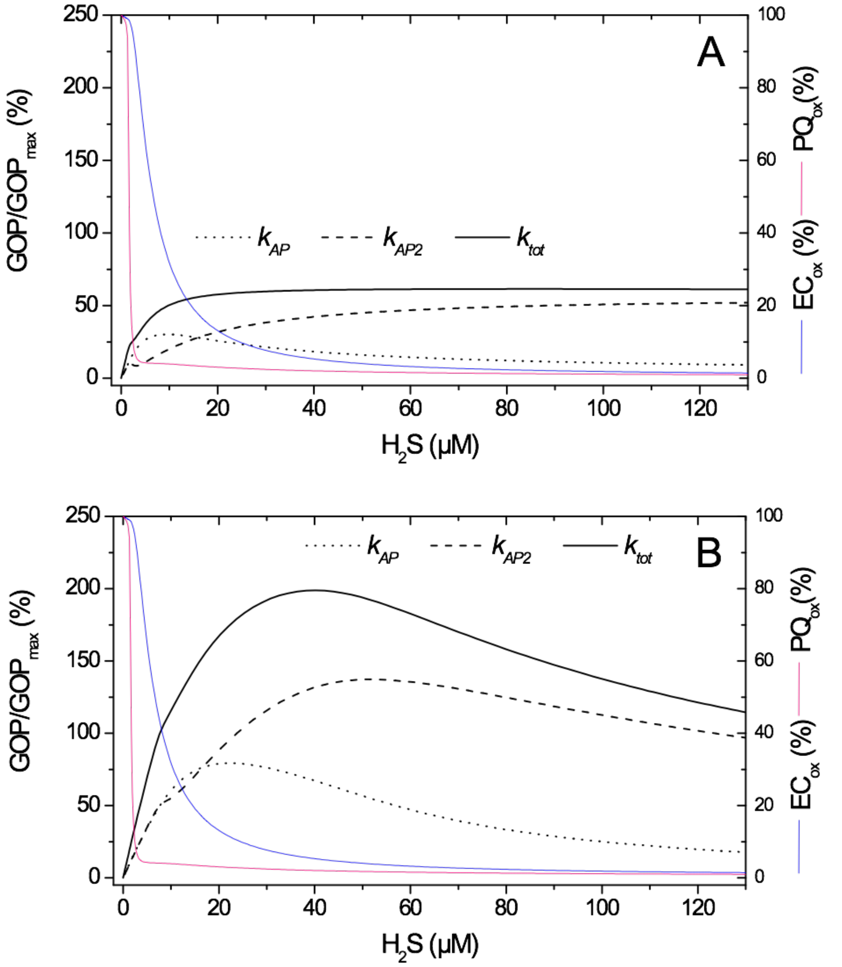

Figure S5. Maximum likelihood phylogeny of SQR sequences. Classification of SQR othologs is based on Gregersen et al., 2011. Bootstrap support values >90 based on 1000 bootstrap samplings are shown for each node.

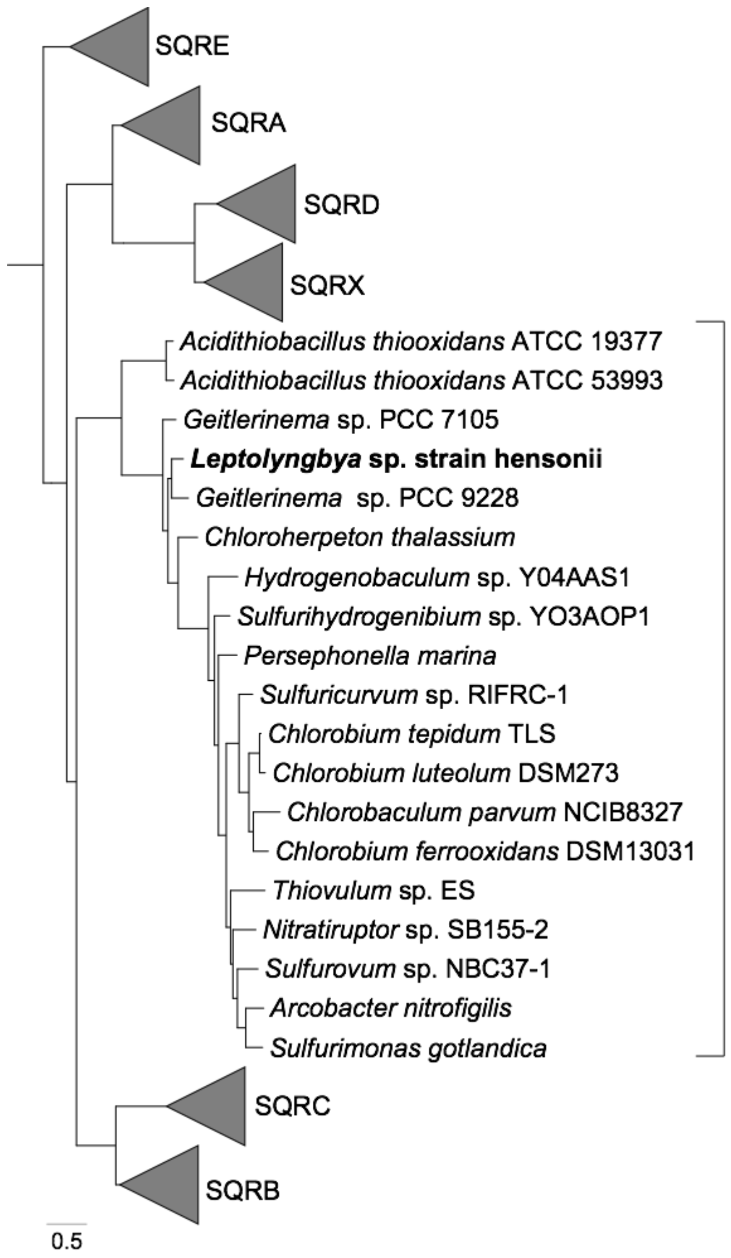

Figure S6. Maximum likelihood phylogeny of HpnP sequences. Black circles indicate bootstrap support values >85 based on 1000 bootstrap samplings. Accession numbers for all sequences are provided in Table S3.

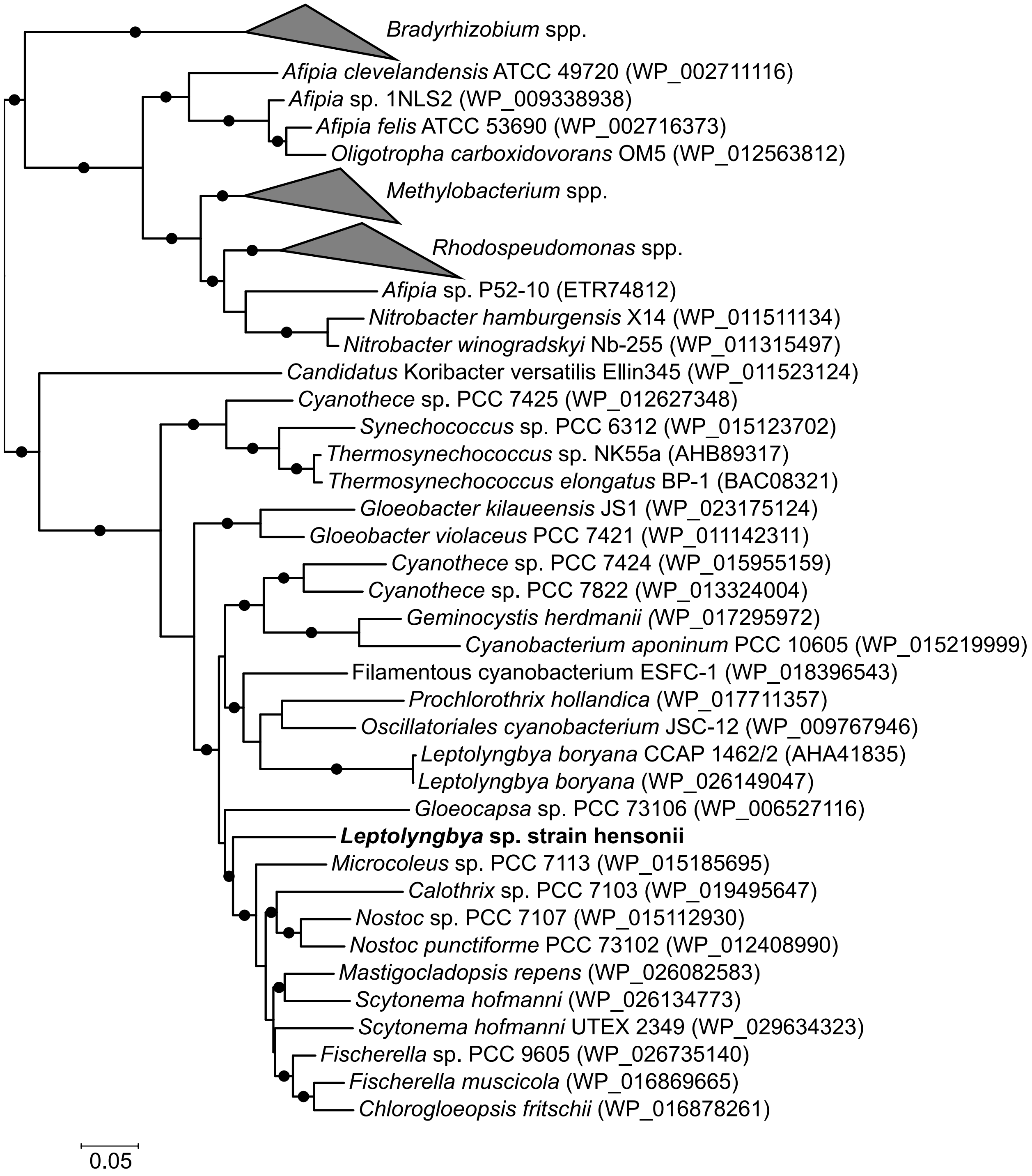

4. SUPPLEMENTAL TABLES

Table S1. Single copy genes used to evaluate genome completeness.

Description	COG	Count
Arginyl-tRNA synthetase	COG0018	1
DNA-directed RNA polymerase subunit alpha	COG0202	1
DNA-directed RNA polymerase subunit beta	COG0085	2
GTP-binding protein YchF	COG0012	1
Histidyl-tRNA synthetase	COG0124	1
Isoleucyl-tRNA synthetase	COG0060	1
Leucyl-tRNA synthetase	COG0495	1
Methionyl-tRNA synthetase	COG0143	1
O-sialoglycoprotein endopeptidase	COG0533	1
Phenylalanyl-tRNA synthetase, alpha subunit	COG0016	1
Protein translocase subunit secY alpha	COG0201	1
Ribosomal protein L1P	COG0081	1
Ribosomal protein L22	COG0091	1
Ribosomal protein L2P	COG0090	1
Ribosomal protein L3P	COG0087	1
Ribosomal protein L4P	COG0088	1
Ribosomal protein L5P	COG0094	1
Ribosomal protein L6P	COG0097	1
Ribosomal protein L11P	COG0080	1
Ribosomal protein L13P	COG0102	1
Ribosomal protein L14P	COG0093	1
Ribosomal protein L15P	COG0200	1
Ribosomal protein L16P	COG0197	1
Ribosomal protein L18	COG0256	1
Ribosomal protein S2P	COG0052	1
Ribosomal protein S3P	COG0092	1
Ribosomal protein S4P	COG0522	1
Ribosomal protein S5P	COG0098	1
Ribosomal protein S7P	COG0049	1

Description	COG	Count
Ribosomal protein S8P	COG0096	1
Ribosomal protein S9P	COG0103	1
Ribosomal protein S11P	COG0100	1
Ribosomal protein S12P	COG0048	1
Ribosomal protein S13P	COG0099	1
Ribosomal protein S15P/S13E	COG0184	1
Ribosomal protein S17P	COG0186	1
Ribosomal protein S19P	COG0185	1
Ribosomal proteinalyl-tRNA synthetase	COG0525	1
Seryl-tRNA synthetase	COG0172	1
Signal recognition particle subunit FFH	COG0541	1

Table S2. Accession numbers of the HpnP sequences included in Figure S3.

Query	Accession Number
Scytonema hofmanni	WP_026134773
Nostoc punctiforme PCC 73102	WP_012408990
Microcoleus sp. PCC 7113	WP_015185695
Mastigocladopsis repens	WP_026082583
Chlorogloeopsis fritschii	WP_016878261
Scytonema hofmanni UTEX 2349	WP_029634323
Fischerella sp. PCC 9605	WP_026735140
Fischerella muscicola	WP_016869665
Nostoc sp. PCC 7107	WP_015112930
Gloeobacter violaceus PCC 7421	WP_011142311
Filamentous cyanobacterium ESFC-1	WP_018396543
Thermosynechococcus elongatus BP-1	BAC08321
Thermosynechococcus sp. NK55a	AHB89317
Oscillatoriales cyanobacterium JSC-12	P_009767946
Prochlorothrix hollandica	WP_017711357
Leptolyngbya boryana	WP_026149047
Cyanothece sp. PCC 7822	WP_013324004
Calothrix sp. PCC 7103	WP_019495647
Synechococcus sp. PCC 6312	WP_015123702
Leptolyngbya boryana CCAP 1462/2	AHA41835
Cyanothece sp. PCC 7425	WP_012627348
Gloeobacter kilaueensis JS1	WP_023175124
Cyanothece sp. PCC 7424	WP_015955159
Cyanobacterium aponinum PCC 10605	WP_015219999
Geminocystis herdmanii	WP_017295972
Gloeocapsa sp. PCC 73106	WP_006527116
Candidatus Koribacter versatilis Ellin345	WP_011523124
Afipia sp. 1NLS2	WP_009338938
Bradyrhizobium sp. ARR65	WP_024508762
Afipia felis ATCC 53690	WP_002716373

Query	Accession Number
Bradyrhizobium sp. Ai1a-2	WP_027581924
Bradyrhizobium sp. BTAi1	WP_012043107
Bradyrhizobium sp. ORS 375	WP_009026937
Bradyrhizobium sp. ORS 278	WP_011925649
Oligotropha carboxidovorans OM5	WP_012563812
Bradyrhizobium oligotrophicum S58	WP_015668065
Bradyrhizobium elkanii	WP_028167187
Afipia clevelandensis ATCC 49720	WP_002711116
Bradyrhizobium sp. STM 3843	WP_008966627
Rhodopseudomonas palustris HaA2	WP_011440614
Bradyrhizobium sp. OHSU_III	WP_024579432
Afipia sp. P52-10	ETR74812
Methylobacterium nodulans ORS 2060	WP_015928577
Bradyrhizobium sp. DFCI-1	WP_021079929
Bradyrhizobium sp. URHA0002	WP_027538041
Bradyrhizobium sp. STM 3809	WP_008962867
Bradyrhizobium elkanii	WP_028331726
Bradyrhizobium elkanii	WP_018270579
Bradyrhizobium elkanii	WP_028350915
Bradyrhizobium sp. ORS 285	WP_006613850
Bradyrhizobium elkanii	WP_028338700
Rhodopseudomonas palustris BisB5	WP_011503969
Methylobacterium sp. 10	WP_027172964
Rhodopseudomonas palustris TIE-1	WP_012497138
Methylobacterium sp. 88A	WP_018044240
Rhodopseudomonas palustris CGA009	WP_011159286
Nitrobacter winogradskyi Nb-255	WP_011315497
Bradyrhizobium sp. th.b2	WP_029082145
Bradyrhizobium sp. Cp5.3	WP_027550404
Bradyrhizobium japonicum	WP_026312822
Bradyrhizobium sp. URHD0069	WP_029585636

Query	Accession Number
Rhodopseudomonas palustris	WP_027277923
Rhodopseudomonas palustris DX-1	P_013501367
Bradyrhizobium japonicum	WP_028157206
Methylobacterium sp. GXF4	WP_007566051
Rhodopseudomonas palustris BisB18	WP_011472175
Methylobacterium sp. 77	WP_026597056
Bradyrhizobium sp. URHA0013	WP_027569005
Methylobacterium sp. 285MFTsu5.1	WP_020096225
Bradyrhizobium japonicum	WP_028169746
Methylobacterium sp. 4-46	WP_012330353
Bradyrhizobium sp. Tv2a-2	WP_024517353
Bradyrhizobium sp. YR681	WP_008144018
Bradyrhizobium japonicum	WP_028135319
Bradyrhizobium sp. WSM2793	WP_026201538
Bradyrhizobium japonicum SEMIA 5079	WP_014496829
Methylobacterium sp. MB200	WP_017484147
Bradyrhizobium sp. CCGE-LA001	EJZ31892
Methylobacterium mesophilicum SR1.6/6	WP_010686897
Bradyrhizobium sp. WSM4349	WP_026233266
Bradyrhizobium sp. WSM1743	WP_027572151
Bradyrhizobium sp. WSM1417	WP_027516850
Bradyrhizobium sp. WSM1253	WP_007593631
Bradyrhizobium sp. S23321	WP_015687471
Bradyrhizobium japonicum	WP_024339740
Bradyrhizobium sp. WSM471	WP_007608072
Nitrobacter hamburgensis X14	WP_011511134
Methylobacterium populi BJ001	WP_012456197
Bradyrhizobium japonicum	WP_028172333
Methylobacterium radiotolerans JCM 2831	WP_012320775
Methylobacterium extorquens DSM 13060	WP_003597531
Bradyrhizobium japonicum	WP_028143224

Query	Accession Number
Methylobacterium extorquens DM4	WP_015824164
Rhodopseudomonas palustris BisA53	WP_011663239
Bradyrhizobium japonicum	WP_028146946
Bradyrhizobium diazoefficiens SEMIA 5080	WP_011085779
Bradyrhizobium sp. WSM3983	WP_027533953

5. SUPPLEMENTAL REFERENCES

Allen JF. (2003). Cyclic, pseudocyclic and noncyclic photophosphorylation: new links in the chain. *Trends Plant Sci* 8:15-19.

Altschul SF, Madden TL, Schäffer AA, Zhang J, Zhang Z, Miller W, et al. (1997). Gapped BLAST and PSI-BLAST: a new generation of protein database search programs. *Nucl Acids Res* 25:3389-3402. (doi: 10.1093/nar/25.17.3389)

Boyd ES, Jackson RA, Encarnacion G, Zahn JA, Beard T, Leavitt WD, et al.(2007) Isolation, characterization, and ecology of sulfur-respiring crenarchaea inhabiting acid-sulfate-chloride-containing geothermal springs in Yellowstone National Park. *Appl Env Microbiol* 73:6669-77. (doi: 10.1128/AEM.01321-07)

Bradford MM. (1976). A rapid and sensitive method for the quantitation of microgram quantities of protein utilizing the principle of protein-dye binding. *Anal Biochem* 72:248–254.

Cole JR, Chai B, Marsh TL, Farris RJ, Wang Q, Kulam SA, et al. (2003). The Ribosomal Database Project (RDP-II): previewing a new autoaligner that allows regular updates and the new prokaryotic taxonomy. *Nucleic Acids Res* 31:442–443.

de Marsac ND, Houmard J. (1988). Complementary chromatic adaptation: Physiological conditions and action spectra. *Methods Enzymol* 167:318-328. (doi: 10.1016/0076-6879(88)67037-6)

Edgar RC. (2004). MUSCLE: multiple sequence alignment with high accuracy and high throughput. *Nucl Acids Res* 19:1792–1797. (doi: 10.1093/nar/gkh340)

Gregersen LH, Bryant DA, Frigaard N-U. (2011). Mechanisms and evolution of oxidative sulfur metabolism in Green Sulfur Bacteria. *Front Microbiol* **2**:116. (doi: 10.3389/fmicb.2011.00116)

Govindjee, Kern JF, Messinger J, Whitmarsh J, Govindjee, Kern JF. (2010). Photosystem II. In: Encyclopedia of Life Sciences (ELS). John Wiley & Sons, Ltd: Chichester, UK. (doi: 10.1002/9780470015902.a0000669.pub2)

Guindon S, Dufayard JF, Lefort V, Anisimova M, Hordijk W, Gascuel O. (2010). New algor ithms and methods to estimate maximum-likelihood phylogenies: Assessing the performance of PhyML 3.0. *Syst Biol* 59:307-421. (doi: 10.1093/sysbio/syq010)

Gutekunst K, Chen X, Schreiber K, Kaspar U, Makam S, Appel J. (2014). The Bidirectional NiFe-hydrogenase in Synechocystis sp. PCC 6803 Is Reduced by Flavodoxin and Ferredoxin and Is Essential under Mixotrophic, Nitrate-limiting Conditions. *J Biol Chem* 289:1930-1937. (doi: 10.1074/jbc.M113.526376)

Jablonsky J, Lazar D. (2008). Evidence for intermediate S-states as initial phase in the process of oxygen-evolving complex oxidation. *Biophys J* 94:2725-2736. (doi: 10.1529/biophysj.107.122861)

Jegerschoeld C, Virgin I, Styring S. (1990). Light-dependent degradation of the D1 protein in photosystem II is accelerated after inhibition of the water splitting reaction. *Biochemistry* 29: 6179–6186. (doi: 10.1021/bi00478a010)

Johnson MP. (2016). Photosynthesis. *Essays Biochem* 60:255–273. (doi: 10.1042/EBC20160016)

Keren N, Berg A, van Kan PJ PJM, Levanon H, Ohad I. (1997). Mechanism of photosystem II photoinactivation and D1 protein degradation at low light: the role of back electron flow. *Proc Natl Acad Sci USA* 94:1579–84.

Klatt JM, Al-Najjar MAA, Yilmaz P, Lavik G, de Beer D, Polerecky L. (2015a). Anoxygenic photosynthesis controls oxygenic photosynthesis in a cyanobacterium from a sulfidic spring. *Appl Environ Microbiol* 81:2025–2031. (doi:10.1128/AEM.03579-14).

Klatt JM, Haas S, Yilmaz P, de Beer D, Polerecky L. (2015b). Hydrogen sulfide can inhibit and enhance oxygenic photosynthesis in a cyanobacterium from sulphidic springs. *Environ Microbiol* 17: 3301–3313. (doi:10.1111/1462-2920.12791)

Kok B, Forbush B, McGloin M. (1970). Cooperation of charges in photosynthetic O2 evolution-I. A linear four step mechanism. *Photochem Photobiol* 11:457–75.

Lane DJ. (1991). 16S/23S rRNA sequencing. In: *Nucleic Acid Techniques in Bacterial Systematics* (eds. Stackebrandt E, Goodfellow, M), John Wiley and Sons, Chichester pp. 115–147.

Lea-Smith DJ, Bombelli P, Vasudevan R, Howe CJ. (2016). Photosynthetic, respiratory and extracellular electron transport pathways in cyanobacteria. *Biochim Biophys Acta-Bioenerg* 1857:247–255. (doi:10.1016/j.bbabio.2015.10.007)

Letunic I, Bork P. (2016). Interactive tree of life (iTOL) v3: an online tool for the display and annotation of phylogenetic and other trees. *Nucl Acids Res* 44:W242-W245. (doi: 10.1093/nar/gkw290)

Messinger J and Renger G (2008) Photosynthetic water splitting. In: Renger G (ed). Primary Processes of Photosynthesis: Basic Principles and Apparatus, Part II Reaction Centers/Photosystems, Electron Transport Chains, Photophosphorylation and Evolution. Royal Society Chemistry: Cambridge, pp 291– 349.

Mullineaux CW. (2014a). Co-existence of photosynthetic and respiratory activities in cyanobacterial thylakoid membranes. *Biochim Biophys Acta-Bioenerg* 1837:503–511. (doi: 10.1016/j.bbabio. 2013.11.017)

Mullineaux CW. (2014b). Electron transport and light-harvesting switches in cyanobacteria. *Front Plant Sci* 5:7. (doi: 10.3389/fpls.2014.00007)

Murata N, Allakhverdiev SI, Nishiyama Y. (2012). The mechanism of photoinhibition in vivo: Re-evaluation of the roles of catalase, α-tocopherol, non-photochemical quenching, and electron transport. *Biochim Biophys Acta-Bioenerg* 1817:1127–1133. (doi: 10.1016/j.bbabio.2012.02.020)

Nagarajan A, Pakrasi HB, Nagarajan A, Pakrasi HB. (2015). Membrane-Bound Protein Complexes for Photosynthesis and Respiration in Cyanobacteria In: Encyclopedia of Life Sciences (ELS). John Wiley & Sons, Ltd: Chichester, UK. (doi: 10.1002/9780470015902.A0001670.PUB2)

Nishiyama Y, Allakhverdiev SI, Murata N. (2006). A new paradigm for the action of reactive oxygen species in the photoinhibition of photosystem II. *Biochim Biophys Acta-Bioenerg* 1757: 742–749. (doi: 10.1016/j.bbabio.2006.05.013)

Peschek GA. (1999). Photosynthesis and Respiration of Cyanobacteria. In: The Phototrophic Prokaryotes. Springer US: Boston, MA, pp 201–209. (doi: 10.1007/978-1-4615-4827-0_24)

Rippka R, Denulles Y, Waterburry JB, Herdman M, Stanier RY. (1979). Generic assignment, strain histories and properties of pure cultures of cyanobacteria. *J Gen Microbiol* **111**: 1–61. (doi: 10.1099/00221287-111-1-1)

Roach T, Krieger-Liszkay AK. (2014). Regulation of Photosynthetic Electron Transport and Photoinhibition. *Curr Protein Pept Sci* 15:351–362. (doi: 10.2174/1389203715666140327105143)

Shevela D, Björn LO, Govindjee. (2013). Oxygenic photosynthesis. In: Razeghifard R (ed). Natural and Artificial Photosynthesis. John Wiley & Sons Inc.: Hoboken, NJ, USA, pp 13-64. (doi: 10.1002/9781118659892)

Soetaert K, Petzoldt T, Setzer RW. (2010). Solving Differential Equations in R: Package deSolve. *J Stat Softw* 33:1–25. (doi: 10.18637/jss.v033.i09)

Vermaas WF. (2001). Photosynthesis and Respiration in Cyanobacteria. In: Encyclopedia of Life Sciences (ELS). John Wiley & Sons, Ltd: Chichester, UK. (doi: 10.1038/npg.els.0001670)

Whitmarsh J, Govindjee. (1999). The Photosynthetic Process. In: Concepts in Photobiology. Springer Netherlands: Dordrecht, pp 11–51.

Zhang P, Eisenhut M, Brandt A-M, Carmel D, Silén HM, Vass I, et al. (2012). Operon flv4-flv2 Provides Cyanobacterial Photosystem II with Flexibility of Electron Transfer. *Plant Cell* 24:1952-71. (doi: 10.1105/tpc.111.094417)
